# Reversible Suppression of Cyclooxygenase 2 (COX-2) Expression *In Vivo* by Inducible RNA Interference

**DOI:** 10.1371/journal.pone.0101263

**Published:** 2014-07-02

**Authors:** Anne K. Zaiss, Johannes Zuber, Chun Chu, Hidevaldo B. Machado, Jing Jiao, Arthur B. Catapang, Tomo-o Ishikawa, Jose S. Gil, Scott W. Lowe, Harvey R. Herschman

**Affiliations:** 1 Department of Medical and Molecular Pharmacology, David Geffen School of Medicine, University of California Los Angeles, Los Angeles, California, United States of America; 2 Department of Biological Chemistry, David Geffen School of Medicine, University of California Los Angeles, Los Angeles, California, United States of America; 3 Cold Spring Harbor Laboratory and Howard Hughes Medical Institute, New York, New York, United States of America; The University of Tennessee Health Science Center, United States of America

## Abstract

Prostaglandin-endoperoxide synthase 2 (PTGS2), also known as cyclooxygenase 2 (COX-2), plays a critical role in many normal physiological functions and modulates a variety of pathological conditions. The ability to turn endogenous COX-2 on and off in a reversible fashion, at specific times and in specific cell types, would be a powerful tool in determining its role in many contexts. To achieve this goal, we took advantage of a recently developed RNA interference system in mice. An shRNA targeting the *Cox2* mRNA 3′untranslated region was inserted into a microRNA expression cassette, under the control of a tetracycline response element (TRE) promoter. Transgenic mice containing the COX-2-shRNA were crossed with mice encoding a CAG promoter-driven reverse tetracycline transactivator, which activates the TRE promoter in the presence of tetracycline/doxycycline. To facilitate testing the system, we generated a knockin reporter mouse in which the firefly luciferase gene replaces the *Cox2* coding region. *Cox2* promoter activation in cultured cells from triple transgenic mice containing the luciferase allele, the shRNA and the transactivator transgene resulted in robust luciferase and COX-2 expression that was reversibly down-regulated by doxycycline administration. *In vivo*, using a skin inflammation-model, both luciferase and COX-2 expression were inhibited over 80% in mice that received doxycycline in their diet, leading to a significant reduction of infiltrating leukocytes. In summary, using inducible RNA interference to target COX-2 expression, we demonstrate potent, reversible *Cox2* gene silencing *in vivo*. This system should provide a valuable tool to analyze cell type-specific roles for COX-2.

## Introduction

Prostanoids modulate a number of complex biological processes, including inflammation and immunity, pregnancy and parturition, cardiovascular function, temperature regulation, neurodegeneration and tumor progression [1]. Prostanoid production is initiated by the cyclooxygenases (COX); enzymes that convert arachidonic acid to the unstable intermediate prostaglandin H2 (PGH_2_). PGH_2_ is then further converted, in cell-specific pathways, to a variety of products that include a number of prostaglandins (PGE_2_, PGF_2a_, PGD_2_, etc.), prostacyclin (PGI_2_) and thromboxane (TXA_2_).

Vertebrate species have two *Cox* genes, *Cox1* and *Cox2*. The COX-1 and COX-2 enzymes are isoforms, similar in structure, that catalyze identical reactions. However, they differ in the regulation of their expression and their biological roles. Most cells constitutively express the COX-1 isoform. Consequently, COX-1 is thought to play a role in homeostatic functions. In contrast, COX-2 is not constitutively expressed in most tissues, but is rapidly induced in many cell types in response to a wide range of mitogens, cytokines and other stimuli, and is often present in elevated levels at sites of inflammation and epithelial cancers [2].

The cyclooxygenases are the targets of nearly all commonly used non-steroidal anti-inflammatory drugs (NSAIDs; e.g., ibuprofen and aspirin). These drugs inhibit COX-1 and COX-2 enzymatic activity, thereby preventing prostanoid production. Prostaglandins are recognized as important modulators of the immune system; COX-2 has been associated with an orchestrating role in both the induction and resolution of acute inflammation [3]. COX-2 is also expressed in sterile inflammation, and is induced by pathogens, but its role in anti-pathogen immune responses is not well understood [4], [5].

The therapeutic effects of NSAIDs and their side effects, as well as studies with COX-2 selective inhibitors (e.g., celecoxib) and with conventional *Cox2* knockout mice suggest roles for COX-2 in a variety of diseases beyond the symptomatic control of pain and fever. Examples of suggested roles for COX-2 aberrant expression include problems in female fertility and parturition [6] and in neurodegenerative diseases such as Alzheimer’s disease and Parkinson’s disease [7]. Considerable research has also been devoted to understanding the role of COX-2 in cancer. COX-2 dependent prostanoids contribute to cell proliferation, and increased COX-2 expression occurs in a wide variety of epithelial cancers [8]. In particular, the beneficial effects of NSAIDs have suggested a major role for COX-2 in colon cancer, where this enzyme is suggested to play modulatory and even causal roles [9], [10].

At what times, and in which subsets of cells, COX-2 expression is required to either promote or suppress tumorigenesis, infection and other biological processes is still unclear. A model system in which spatial, temporal and reversible loss of COX-2 function could be achieved would, therefore, be of great value in understanding the roles COX-2 plays in normal and pathophysiological conditions.

One way cells achieve targeted modulation of gene expression is through RNA interference (RNAi). RNAi is an endogenous mechanism in which short hairpin RNA (shRNA) species modulate targeted transcripts. In mammals, naturally occurring shRNAs are usually embedded within a microRNA (miRNA) backbone [11]. The miRNA is processed to a short hairpin RNA consisting of a sequence of 21–29 nucleotides, a short loop region and the reverse complement of the 21–29-nucleotide region. This short RNA molecule folds back on itself to form a hairpin structure, which is cleaved into double-stranded RNAs by ‘Dicer’, an endogenous nuclease. After nuclease digestion the RNA silencing complex recognizes the shRNA and guides it to complementary mRNA(s), thereby targeting them for destruction [12], [13]. In this manner, RNAi regulates sequence-specific mRNA destruction.

Investigator-initiated experimental RNAi gene silencing has advantages over traditional gene targeting methods, since RNAi agents act without modifying the target gene. Cullen et al. [14] demonstrated they could use a scaffold to embed and subsequently express a synthetic shRNA sequence targeting a gene of interest. Using the microRNA30 (miR30) precursor RNA as a template, they substituted miR30 stem sequences with designed shRNAs, and showed effective target gene inhibition [15]. When shRNAs are expressed from a tetracycline response element (TRE) promoter in cells, along with a tetracycline transactivator (tTA or rtTA) protein, the shRNA can be reversibly expressed in response to doxycycline (DOX) presence or absence [16], [17]. Both a ‘tet-off’ system, in which the tet-transactivator (tTA) protein activates the TRE promoter but is inhibited by the administration of DOX and a ‘tet-on’ system, in which the reverse tet-transactivator (rtTA) is latent until activated by DOX, are in common usage for reversible regulation of gene expression [18]. Many mouse lines have been developed that express the tTA (tet off) or rtTA (tet-on) transactivators from tissue specific promoters, allowing temporal and reversible control of gene expression in specific cell types [19].

The ability to reversibly control the location, timing and levels of COX-2 expression would facilitate the elucidation of the many and varied COX-2 roles in normal physiology and disease. In this study we describe the development of a mouse model in which DOX-regulated expression of a COX-2 targeted shRNA makes it possible to inactivate endogenous *Cox2* gene expression, providing cell-specific, reversible elimination of COX-2 expression and function.

## Materials and Methods

### COX-2 shRNA design and cloning

**Figure 1:**
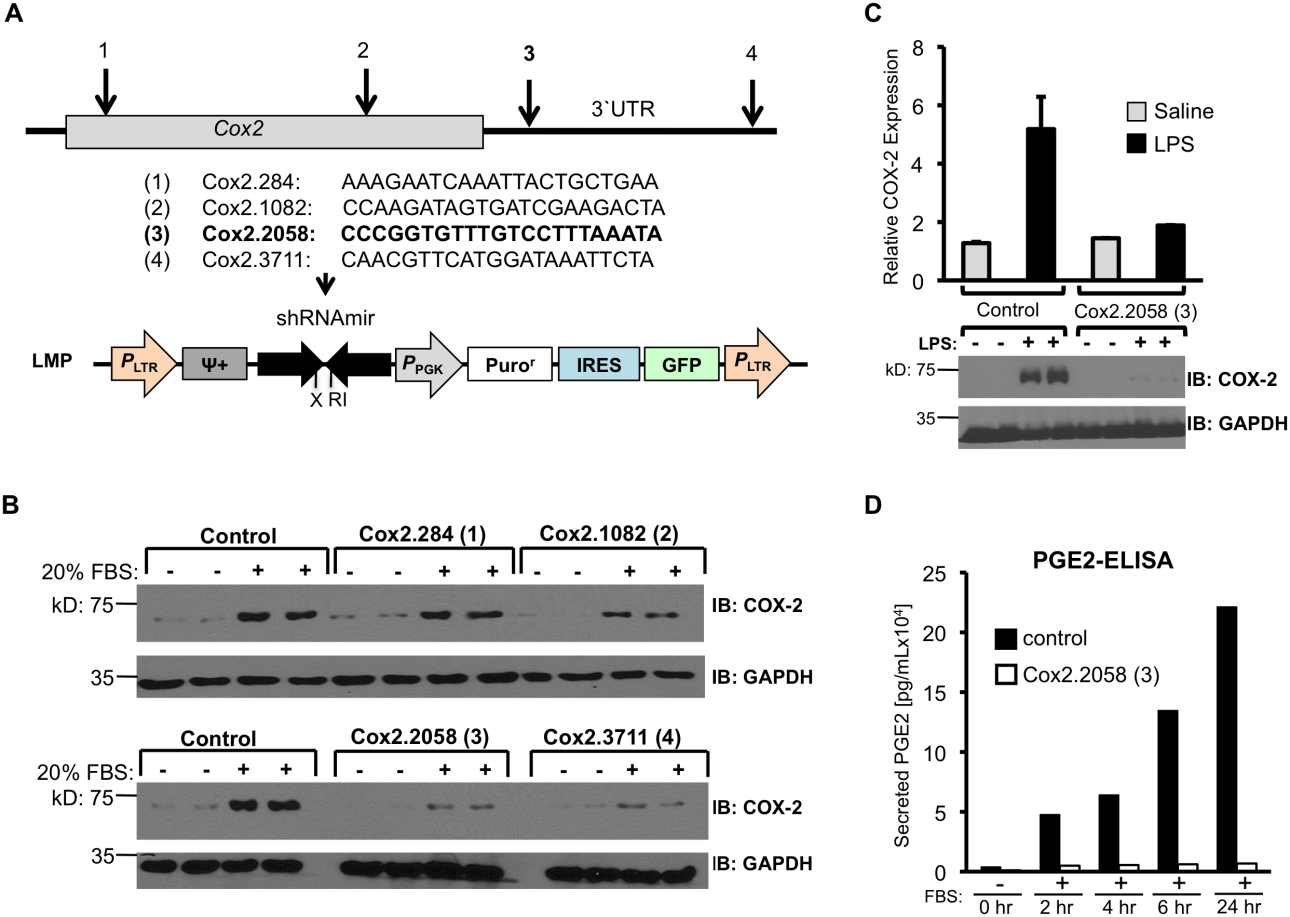
Identifying small inhibitory RNAs for COX-2. (**A**) Schematic representation of the *Cox2* transcript, indicating target areas of four designed shRNAs. The COX-2 protein-coding region is indicated as a filled box, 5′ and 3′ untranslated mRNA as solid lines. Four 22-nucleotide shRNAs for *Cox2* were designed; (1) Cox2.284, (2) Cox2.1082, (3) Cox2.2058 and (4) Cox2.3711, the number reflecting the first nucleotide position of the target sequence in the mRNA transcript. The shRNA sequences were then converted into cloning templates and ligated into the LMP retrovirus vector. This vector contains an *Xho*I*/Eco*R1 cloning site for shRNAs within a miR30 backbone (shRNAmir). The LMP construct is shown as it appears after integration; shRNAs are constitutively expressed from the 5′LTR promoter. The LMP retrovirus also encodes a puromycin-resistance gene for selection and GFP as a fluorescent marker. (**B**) NIH3T3 cells were transduced at a low MOI with each of the four LMP vectors containing miR30-based shRNAs that target the *Cox2* transcript, or with a control luciferase-targeted shRNA. Retrovirus-transduced cells were selected for integrated provirus by culturing in puromycin. Puromycin-selected cell populations were shifted overnight to 1% serum, then treated with 20% serum for 6 hours. Cell extracts were immunoblotted for COX-2 and GAPDH. (**C**) RAW264.7 cells stably transduced either with the luciferase shRNA LMP vector or with the LMP vector encoding *Cox2*-targeted Cox2.2058 shRNA were stimulated with LPS (50 ng/mL) or with saline for four hours. Cell extracts were prepared and analyzed for COX-2 protein and GAPDH. The quantified COX-2 signal was normalized against GAPDH. (**D**) PGE_2_ accumulation in the media of serum-stimulated NIH3T3 cells expressing shRNA Cox2.2058 or the control luciferase shRNA. NIH3T3 cells expressing the two shRNAs were shifted from media containing 1% FBS to 20% FBS and, at times shown, media samples were assayed for PGE_2_ levels.

Potential gene silencing short hairpin RNAs (shRNAs) targeting COX-2 were designed as described [20]. Predicted shRNAs were screened against a series of sensor exclusion criteria and cross-checked against a transcript database to exclude sequences with similarity to ‘off-target’ genes [20]. Four 22-mer predicted shRNAs; Cox2.284, Cox2.1082, Cox2.2058 and Cox2.3711 were identified; numerical designations reflect the first nucleotide position of the targeted mouse *Cox2* mRNA sequence (Fig. 1A). These shRNA sequences, and their corresponding sense strand predictions, were synthesized as 97 mers and cloned into the miR30 shRNA backbone as described previously [21]. These sequences comprise the common and gene-specific stem and 19 bp loop of the miR30-context to create miR30-adapted shRNAs specific for *Cox2.*

### Retrovirus vector preparation and shRNA testing

The LMP retroviral vector, a murine stem cell virus (MSCV)-based vector contains unique *Xho*I and *Eco*RI sites within a miR30-shRNA expression cassette, driven by the viral 5′LTR promoter ([17], [20] and Fig. 1A). The vector also encodes PGK promoter-driven puromycin resistance and green fluorescent protein sequences, separated by an internal ribosome entry site (IRES). The four *Xho*I*/Eco*RI COX-2*-*shRNAs were cloned into the LMP vector, and retroviral stocks for the four COX-2*-*shRNAs and a retrovirus containing a control shRNA targeting the firefly luciferase coding sequence were generated by three plasmid co-transfection using a VSV-G envelope plasmid in HEK293 cells (ATCC).

To test the ability of the four COX-2 shRNAs to block COX-2 induction at single copy integrated levels, NIH3T3 mouse embryonic fibroblast cells and RAW264.7 (ATCC) mouse monocytic leukemia cells were transduced with a viral multiplicity of infection (MOI) resulting in <1% GFP-positive cells. Cells selected with puromycin (2.5 µg/ml) were used to test COX-2 induction 3–5 days later. NIH3T3 cells were cultivated in DMEM-supplemented with 10% fetal bovine serum (FBS) and 1% penicillin-streptomycin at 37°C in 5% CO_2_. RAW264.7 cells were maintained in RPMI 1640 medium with 10% FBS and 1% penicillin-streptomycin. COX-2 was induced in RAW264.7 cells by adding bacterial lipopolysaccharide (LPS, Sigma) for four hours. NIH3T3 cells were starved with 1% FBS over night before shifting to medium with 20% FBS for six hours to induce COX-2 expression. Cells were washed with PBS and lysed directly in the culture dish with Passive Lysis Buffer (Promega). For prostaglandin E2 (PGE_2_) analyses culture supernatants were harvested, snap frozen in liquid nitrogen, and assayed collectively by ELISA (Cayman Chemical).

### Generation of the TRE-shRNA transgenic mouse

Animal experiments were conducted according to guidelines of the UCLA Animal Care Committee and the Cold Spring Harbor Laboratory Animal Facility. Protocols were subject to ethical review and approval by the UCLA Animal Research Committee (Protocol No. 2010-074-01). Mice were generated using the Flp/FRT recombinase-mediated cassette exchange (RMCE) strategy, using ‘KH2’ C57BL/6; SJL ES cells that contain an frt-hygro-pA ‘homing’ cassette downstream of the *ColA1* gene on mouse chromosome 11 [22]–[24]. Site-specific integration into the homing cassette is achieved by FLPe recombinase-mediated recombination between the FRT-sites in the *ColA1* locus on chromosome 11 and in the targeting vector. The targeting vector, pCol-TGM, contains a GFP open reading frame immediately downstream of the TRE promoter, followed by the miR30-based shRNA expression cassette. The Cox2.2058 shRNA in the LMP shRNA expression cassette was cloned into the miR30 backbone of this targeting vector at the single *Xho*I*/Eco*RI site. The pCol-TGM targeting vector containing the Cox2.2058 shRNA and a plasmid expressing Flpe-recombinase (pCAGs-Flpe) were then co-electroporated into KH2 ES cells. Flpe-mediated recombination confers hygromycin resistance. Sequencing confirmed Cox2.2058 shRNA in Hygromycin-resistant ESC clones. Transgenic mice were then generated using tetraploid embryo complementation, to give rise to mice that are derived directly from the targeted ES cells [25]–[27].

To elicit doxycycline (DOX) inducible expression from the TRE promoter we used the CAGGs-rtTA3 (4288) transactivator mouse line [24], which expresses rtTA3 from the chicken beta-actin/CMV (CAGGs) promoter. CAGGs-rtTA3 (termed “CAG-rtTA3” or “C3”) and TG-Cox2.2058 (termed “shCox-2”) mice were crossed to produce double transgenic TG-Cox2.2058/CAG-rtTA3 (shCox2/C3) mice. Offspring were genotyped by genomic DNA PCR for the shCox2 [ColArev45 primer [20] and shCox2 forward primer: 5′-AAA GGA CAA ACA CCG GAT GC-3′] and C3 alleles [24]. To guarantee that all shCox2/C3 mice carry only a single copy of the shCox2 or C3 transgenes, mouse strains were maintained separately and only crossed when generating double transgenic mice.

### Generation of a targeted luciferase knockin mouse with the *Cox2* 3′untranslated region (3′UTR)

**Figure 2:**
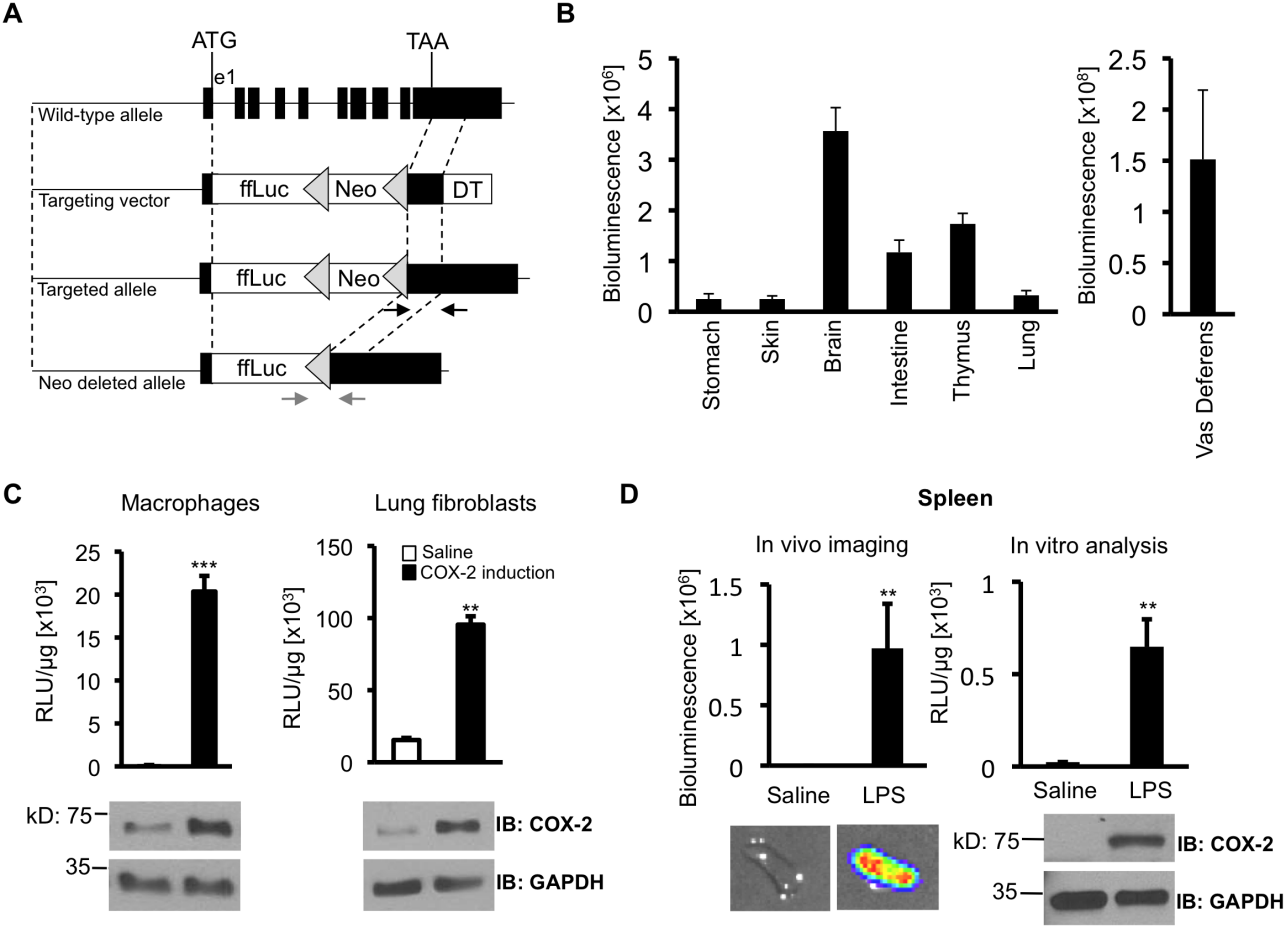
Construction of *Cox2^tm2Luc/+^* a mouse strain in which firefly luciferase replaces the *Cox2* coding region. (**A**) Schematic representation of the wild-type *Cox2* allele and the targeting strategy to create the *Cox2^tm2Luc^* knockin allele. The firefly luciferase coding region (ffLuc), PGK-neo (neo) selection cassette, and PGK-DT (DT) selection cassette in the targeting vector are shown as open boxes. Grey triangles depict loxP sites. Homologous recombination was confirmed by PCR (black arrows) and Southern blot analysis in ES cells. The neomycin-resistance cassette was deleted by Cre recombinase expression, resulting in the ‘neo-deleted’ allele. Deletion was confirmed by PCR (grey arrows). *Cox2* gene sequences are replaced by the firefly luciferase coding region between the ATG translational start site located at the end of exon 1 (e1) and the TAA *Cox2* stop codon located on exon 10. There are no modifications of the untranslated 5′UTR and 3′UTR either upstream of the ATG or downstream from the TAA. (**B**) Unstimulated luciferase activity in isolated *Cox2^tm2Luc/+^* tissues. Luciferase activity was quantified by *ex vivo* bioluminescent imaging. Data are means +/− SD (n = 4). (**C**) COX-2 and luciferase induction in primary cells isolated from *Cox2^tm2Luc/+^* mice. Bone marrow macrophage cultures were stimulated with LPS (50 ng/mL) for four hours. Lung fibroblast cultures were stimulated with 20% serum for six hours. Cell extracts were analyzed for luciferase enzymatic activity and COX-2 protein. Luciferase activity is displayed as relative light units (RLU) per microgram protein. Data are means +/− SD (**, *p*<0.01, ***, *p*<0.001, n = 3). (**D**) Interferon gamma and endotoxin (IFNγ/LPS) COX-2 and luciferase induction in the spleens of heterozygous *Cox2^tm2Luc/+^* mice. Four mice were injected i.p. with IFNγ, (1 µg/mouse) and two hours later with LPS (3 mg/kg) or saline. After 6 hours, mice were euthanized, spleens were excised and luciferase bioluminescence was quantified by bioluminescent imaging (left panel). Luciferase enzymatic activity and COX-2 protein levels were measured in extracts (right panel). Data are means +/− SD (**, p<0.01).

We followed a procedure similar to that used in the construction of the *Cox2^fluc^* knockin allele, in which the firefly luciferase (ffLuc) coding region and an SV40 3′UTR are substituted for the COX-2 coding region and 3′UTR [28]. The targeting vector contains a 6.7 kb genomic region upstream of the *Cox2* ATG initiation codon, the firefly luciferase coding region placed at the *Cox2* gene initiating ATG codon, a floxed neo cassette for positive selection, a 1 kb genomic region downstream of the *Cox2* termination codon, and the diphtheria toxin cassette for negative selection (Fig. 2A). This plasmid was generated by recombineering, using pCOXLuc-DT created previously [28], which contains the 5′ *Cox2* genomic region and the ffLuc gene, and pCOXlucNeo^floxed^COX_3UTR_DT which contains the remaining elements described above. The final targeting vector was confirmed by DNA sequencing. To generate *Cox2* knockin mice, the targeting vector was linearized by *Not*I digestion and electroporated into LW1 embryonic stem (ES) cells. ESC clones containing the *Cox2^luc-fl^* ^o*xed-ne*o^ allele were screened by PCR using the primer pair PGKRa (5′-CTAAAGCGCATGCTCCAGACT-3′ targeting the PGK promoter) and NeoPCR-REV (5′-TAAAGTGACCACGAGAAACGGA-3′ targeting the *neoB* gene). Homologous recombination was verified by Southern blotting using a 175 bp SacI/ApaI fragment within the Cox2 5′-UTR to probe the *Pvu*II/*Nhe*I-digested mouse genomic DNA. ESC clones were injected into C57BL/6 blastocysts to generate chimeric mice. One germline transmission founder was obtained. This mouse was crossed with a Cre transgenic mouse to generate *Cox2* heterozygous mice. Complete excision of the floxed neo cassette was confirmed by PCR (Luc-F4 5′-GCT GGG CGT TAA TCA GAG AG-3′, Lox-R2 5′-CCA AGC TAT CGA ATT CCT GC-3′) and Southern blot. The targeted *Cox2* allele is referred to as *Cox2^tm2Luc^* (‘*Cox2* targeted mutation 2 Luciferase’) or ‘Luc’.

The *Cox2^tm2Luc^* allele was detected in cells and in mice by PCR: Luc1 primer, 5′-CCA GGG ATT TCA GTC GAT GT-3′ and Luc2 primer; 5′-CGC AGT ATC CGG AAT GAT TT-3′). *Cox2* wild type primers: CoxF1, 5′-AAT TAC TGC TGA AGC CCA CC-3′; E4R2: 5′-AGA AGG CTT CCC AGC TTT TGT AAC C-3′.

### Isolation and culture of mouse primary bone marrow macrophages and fibroblasts

#### Macrophages

Bone marrow macrophages were isolated from the tibia, femur and pelvis, using standard procedures. Cells were washed, depleted of red blood cells, plated into RPMI 1640 medium (Gibco/Invitrogen) supplemented with 10% FBS, 1% Penicillin/Streptomycin, 10 mM Hepes and 0.01 µg/mL mouse macrophage colony stimulating factor (M-CSF, Gibco/Invitrogen), and cultured for 5 days at 37°C in 10% CO_2_. All experiments were performed in the following three days. For *Cox2* gene activation, cells were treated with LPS (50 ng/mL) for 4 hours in the presence or absence of DOX (Clontech), as described in Results and Figure Legends.

#### Fibroblasts

Primary lung and skin fibroblasts were isolated as previously described [29]. Tissues were collected, cut into small pieces, treated with liberase (Roche) for 60 minutes, and then cultured at 37°C in 5% CO_2_ for 5 days in DMEM/F12 medium (Gibco/Invitrogen) supplemented with 15% FBS and 1% Penicillin/Streptomycin, to allow fibroblasts to migrate from the fragments. Remaining tissue pieces were removed and the medium was changed to EMEM (Gibco/Invitrogen) supplemented with 15% FBS, 1% Penicillin/Streptomycin, non-essential amino acids and sodium pyruvate. COX-2 and luciferase expression was induced and analyzed as described above for retrovirus transduced NIH3T3 cells. DOX was added to the media at times, concentrations and durations indicated in Results and Figure Legends. DOX was changed daily.

### *Cox2* gene activation *in vivo*

#### Cox2 gene activation by systemic interferon gamma and endotoxin

**Systemic** *Cox2* activation by IFN-γ and LPS for triple transgenic shCox2/C3/Luc+ mice was performed as described previously [28]. Control mice were injected with saline. Six hours after LPS injection the mice were euthanized. Tissues were rapidly removed, placed in culture dishes and used for luciferase-dependent bioluminescence imaging. The tissues were then snap-frozen, and used subsequently for *in vitro* luciferase enzyme activity quantification and for COX-2 Western blotting.

#### Cox2 gene activation in the mouse paw by zymosan

Triple transgenic shCox2/C3/Luc+ mice were fed a DOX-free diet or a DOX (625 mg/kg)-containing diet (Harlan Tek) for 12 days. Inflammation was induced by sub-plantar Zymosan injection into the left hind paw, as described previously [28]. Saline injection into the right hind paw was used as a control. At the indicated time points mice were anesthetized with isoflurane and imaged non-invasively for GFP-dependent fluorescence as surrogate for shRNA expression and for luciferase-dependent bioluminescence.

#### Cox2 gene activation in skin by 12-O-tetradecanoylphorbol-13-acetate (TPA)

Mice were anesthetized briefly by isoflurane inhalation. A small area of the dorsal skin was exposed by shaving and TPA (LC Laboratories, 5 µg in acetone) was applied. Twenty-four hours later mice were imaged non-invasively for GFP-dependent fluorescence to analyze shCox2 expression and for luciferase-dependent bioluminescence. Alternatively, mice were euthanized and skin was collected and processed for Western blotting.

### Bioluminescence and fluorescence imaging

For bioluminescence *in vivo* imaging, mice were anesthetized with isoflurane, shaved if applicable, injected i.p. with D-luciferin (125 mg/kg) and placed into the imaging chamber of an IVIS imaging system (Xenogen). For *ex vivo* tissue imaging, mice were euthanized and tissues were rapidly excised, placed on culture dishes, covered with D-luciferin (15 mg/mL) solution and imaged. Bioluminescence emissions were collected over 3 minute periods at 5-minute intervals. Whole body and *ex vivo* tissue images were acquired repeatedly until the maximum peak of photon number was confirmed during the 3 minute scans. Data at the 3-minute time point that gave the highest photon number were used for further analysis, using Living Image software (Perkin Elmer). For bioluminescence quantification, a region of interest (ROI) was drawn manually and bioluminescence was recorded as radiance (peak photon/sec/cm^2^/sr).

*In vivo* fluorescent images were obtained at 465 nm excitation and 520 nm emission wavelengths. Fluorescence was quantified by drawing ROIs and displayed as mean fluorescent intensity.

### Luciferase activity assays

Cultured cells and tissues were washed with PBS and homogenized in Passive Lysis Buffer (Promega). Debris was removed by centrifugation and luciferase activity was determined in extracts, using the Luciferase Assay System (Promega) and Luminat LB9501 instrumentation. Luciferase activity in cell/tissue extracts was normalized to protein content determined by Bradford assay, and displayed as relative light units per µg protein (RLU/µg).

### Western blotting

The following antibodies were used for Western blotting: GFP, rabbit polyclonal IgG, Abcam, ab290, 1∶2000; glyceraldehyde 3-phosphate dehydrogenase (GAPDH), rabbit polyclonal IgG, Santa Cruz, sc-25778, 1∶1000; COX-2 (in skin), rabbit polyclonal IgG, Cayman, 160106, 1∶100; COX-2 (all other tissues and cells), rabbit polyclonal IgG, Santa Cruz, sc-1747-R, 1∶1000. Extracts of cells or tissues that were previously lysed in Passive Lysis Buffer were mixed with SDS sample buffer and boiled prior to separation on SDS-PAGE gels. For Western blot analysis of skin, tissue was homogenized in RIPA buffer containing a proteinase inhibitor cocktail (Santa Cruz, sc-24948), sonicated and incubated at 4°C for 20 minutes on a rocking platform. Cell debris was removed by centrifugation and protein content was determined by Bradford assay.

Proteins (40–80 µg) were separated on 10% SDS-PAGE gels and transferred onto nitrocellulose membranes. The membranes were blocked with 4% milk protein in PBS/0.1% Tween-20, probed with primary antibodies in the same buffer over night at 4°C, then incubated with anti-rabbit HRP-conjugated secondary antibody (Santa Cruz, sc-2004, 1∶5000) for one hour at room temperature. Proteins were visualized on autoradiographic film using ECL reagent (Pierce). For quantification, membranes were scanned with Typhoon instrumentation. Results were graphed as volume densities relative to the GAPDH signal.

### Histology and immunohistochemical staining

For immunohistochemistry of COX-2 and GFP, skin samples were fixed overnight in 10% buffered formalin, then embedded in paraffin. Skin sections (5 µm) were de-paraffinated and treated for antigen retrieval with the citrate buffer heat method. Endogenous peroxidases were quenched with 3% hydrogen peroxide and sections were blocked in 5% normal donkey serum/PBS for one hour before overnight incubation with rabbit anti-COX-2 antibody (1∶25; Cayman, 160106) or rabbit anti-GFP antibody (1∶5000; Abcam, ab290) at 4°C. Slides were washed and incubated with biotin-labeled goat anti-rabbit secondary antibody (1∶250; Jackson Laboratories). Signal was developed using the ABC and DAB vector kits (Vector Laboratories). Sections were counterstained with hematoxylin.

To measure leukocyte infiltration, sections were stained with hematoxylin and eosin and the numbers of infiltrating leukocytes were counted in six fields per section in a blinded fashion. Images were obtained with an inverted microscope (Nikon, Eclipse 2000 TE) using NIS Elements imaging software.

### Statistics

Data are expressed as means +/− S.D. (standard deviation). Data were compared between groups with the unpaired Student’s *t* test or one-way analysis of variance as appropriate. *p* values less than 0.05 were considered significant. All experiments were performed a minimum of three times with a minimum of triplicate samples.

## Results

### Identifying small hairpin RNAs that can block COX-2 expression

Using improved prediction methods for the design of miR30-based shRNAs [20], we identified four 22-mer guide strand sequences; Cox2.284 (1), Cox2.1082 (2), Cox2.2058 (3) and Cox2.3711 (4) (Fig. 1A), complementary to the *Cox2* coding region (1 and 2) or the *Cox2* 3′-UTR sequence (3 and 4). Guide and complementary sense strand sequences, were embedded into cloning templates as described [20]. Appropriate products carrying the *Xho*I*/Eco*RI restriction sites at their ends and comprising the common and *Cox2-*specific stem sequences and the 19 bp loop were used to create miR30-adapted shRNAs.

Since shRNA transgenic mice will harbor only one copy of the shRNA expression cassette, it is necessary to identify shRNAs that efficiently silence COX-2 expression when expressed from a single genomic locus. To identify appropriate shRNAs, each cloning template containing a COX-2 shRNA sequence was ligated into LMP, a retroviral miR30-shRNA expression vector in which miRNA-based shRNA (shRNAmir) expression is driven from the viral 5′LTR promoter (Fig. 1A). The vector also contains a PGK promoter-driven puromycin resistance gene and a green fluorescence protein (GFP) gene to select and identify transduced cells (Fig. 1A, lower illustration).

To test the candidate shRNAs for suppression of COX-2 expression when expressed from a single gene copy, NIH3T3 cells were transduced with the four LMP vectors at a very low multiplicity of infection (MOI). Flow cytometry of the transduced cell populations demonstrated that only ∼1% of the cells expressed GFP (Supplementary Fig. 1A), suggesting transduced cells contained only a single viral genome. Subsequent puromycin selection ensured expansion only of transduced cells, resulting in a mixed cell population of single integrants. Southern blot analysis (Fig. S1B–D) confirmed multiple integration sites in retrovirus transduced, antibiotic-selected cell populations.

The *Cox2* gene can be induced in NIH3T3 cells by increasing the serum concentration in the medium. The abilities of the four *Cox2* specific shRNAs to block COX-2 protein expression were examined by stimulating the four retrovirus-transduced NIH3T3 cell populations with medium containing 20% serum for six hours, then analyzing cell extracts for COX-2 protein content (Fig. 1B). As expected, cells transduced with a vector encoding a control shRNA against luciferase demonstrated substantial COX-2 protein induction. Cells transduced with COX-2 shRNAs 1 and 2 also expressed substantial COX-2 protein in response to 20% serum; these shRNAs did not have a significant effect on COX-2 expression at single-copy conditions. In contrast, cells transduced with COX-2 shRNAs 3 and 4 expressed only small amounts of COX-2 protein in response to serum stimulation. We chose COX-2 shRNA 3 (Cox2.2058) for further studies, as its target location in the mRNA should make it effective in silencing COX-2 expression from both the short and long *Cox2* gene transcripts [30].

To confirm COX-2 knockdown by Cox2.2058 in a different cell type, LMP vector transduction and puromycin selection were repeated in RAW264.7 cells, a murine monocyte cell line. RAW264.7 cells transduced with the control luciferase shRNA vector express substantial COX-2 protein in response to bacterial lipopolysaccharide (LPS, 50 ng/mL), compared to saline-treated cells (Fig. 1C). In contrast, RAW264.7 cells transduced with Cox2.2058 did not express COX-2 protein above baseline values when treated with LPS. Western blot data quantification indicated over 90% reduction of LPS-induced COX-2 expression (Fig. 1C).

To demonstrate functional inactivation of the *Cox2* transcript by Cox2.2058, prostaglandin E2 (PGE_2_) production was examined in serum-stimulated NIH3T3 cells expressing either the control luciferase shRNA or Cox2.2058. Serum-stimulated cells expressing the control shRNA secrete continuously increasing amounts of PGE_2_ into the culture medium (Fig. 1D). In contrast, *Cox2*-targeted Cox2.2058 expression completely eliminated serum-induced PGE_2_ accumulation. In summary, shRNA 3 (Cox2.2058) was highly effective in suppressing COX-2 expression and activity when expressed at a single copy per cell.

#### A reporter mouse in which the endogenous *Cox2* coding region is replaced with the firefly luciferase coding region

To test the effects of the COX-2 shRNA Cox2.2058 on COX-2 expression it would be useful to have a reporter system that could be non-invasively and repeatedly monitored in individual mice. We previously constructed a knock-in mouse, *Cox2^fLuc/+^*, in which firefly luciferase is expressed from the endogenous *Cox2* gene [28], and have used this mouse to monitor expression from the *Cox2* gene in a variety of contexts [28], [31]–[33]. However, the *Cox2^fLuc^* allele contains an SV40 3′UTR substituted for the endogenous *Cox2* 3′UTR. Because Cox2.2058 targets the 3′UTR of the *Cox2* mRNA, we constructed a new knock-in mouse in which the luciferase coding region is also driven by the endogenous *Cox2* promoter, but in which the luciferase coding sequence is followed by the endogenous *Cox2* 3′UTR. Using this mouse, the efficacy of shRNA-mediated *Cox2* knockdown, and its reversibility can be evaluated by noninvasive imaging of *Cox2* promoter-driven luciferase activity.

We employed a knockin strategy whereby the firefly luciferase (ffluc) coding region and a PGK-neomycin selection cassette in the targeting vector were inserted into the *Cox2* locus by homologous recombination in ES cells (Fig. 2A). Immediately downstream from the neo selection cassette for selection of ES cells containing the targeted allele, the targeting vector contains the *Cox2* translational stop codon (TAA), followed by the *Cox2* gene 3′UTR of exon ten and a diphtheria toxin cassette. After selection of ES cells containing the targeted allele, the neo selection cassette was excised by transient Cre expression, resulting in the final neo-deleted, ffLuc-expressing allele. This second *Cox2*-targeted firefly luciferase knock-in allele was termed ‘targeted mutation 2 luciferase’ (tm2Luc).

To characterize *Cox2^tm2Luc/+^* mice we first determined which organs demonstrate detectable luciferase gene expression in untreated mice. Luciferase expression was examined by bioluminescent imaging of tissues dissected from *Cox2^tm2Luc/+^* mice, and quantified from the optical imaging data. The highest constitutive luciferase expression from the *Cox2* gene occurred in the vas deferens (Fig. 2B, right panel), consistent with what has been reported previously [34]. Substantial luciferase expression was also observed in the brain, throughout the gastrointestinal tract, in the thymus, and to lesser extent in stomach, skin and lungs (Fig. 2B, left panel). Luciferase activity was low or undetectable in all other organs examined, including pancreas, kidney, liver, spleen, and heart. The luciferase expression pattern confirms published results for COX-2 expression obtained by immunohistochemistry and Western blotting, and by our previous results with the *Cox2^fLuc/+^* mouse [28], [31], [34], [35].

To further characterize luciferase and COX-2 expression in tissues from heterozygous *Cox2^tm2Luc/+^* mice, bone marrow macrophages and lung fibroblasts from mice were harvested, cultured and appropriately stimulated to induce *Cox2* expression. Cell extracts were assayed both for luciferase enzymatic activity and for COX-2 protein (Fig. 2C). LPS (50 ng/mL)-treated macrophages showed parallel increases in luciferase activity and COX-2 protein expression. Serum-stimulated lung fibroblasts also showed similar concomitant luciferase enzymatic activity and COX-2 protein accumulation.

The final set of experiments validating the *Cox2^tm2Luc/+^* mouse evaluated coordinated luciferase and COX-2 expression *in vivo*. Systemic interferon gamma (IFN-γ) and endotoxin (LPS) injection induces robust COX-2 induction in spleen, lung, heart and liver [28]. *Cox2^tm2Luc/+^* mice were injected first with IFNγ 1 µg/mouse), and two hours later with LPS (3 mg/kg). Six hours after LPS injection organs were removed, exposed to luciferin substrate, and imaged to measure bioluminescence. Substantially enhanced bioluminescence was observed in spleen, liver, heart and lung tissues of [IFN-γ + LPS] injected *Cox2^tm2Luc/+^* mice, compared to saline injected control mice (Fig. 2D, left panel and Fig. S2). After imaging, tissues were homogenized and extracts were analyzed both for luciferase enzymatic activity and for COX-2 expression (Fig. 2D, right panel and Fig. S2). IFN-γ+LPS induced both luciferase and COX-2 protein in the tissues examined; demonstrating luciferase induction from the *Cox2^tm2Luc^* allele can be used to determine COX-2 expression in heterozygous *Cox2^tm2Luc/+^* mice.

#### Generation of a tetracycline-inducible short hairpin RNA for COX-2 inhibition in mice

**Figure 3:**
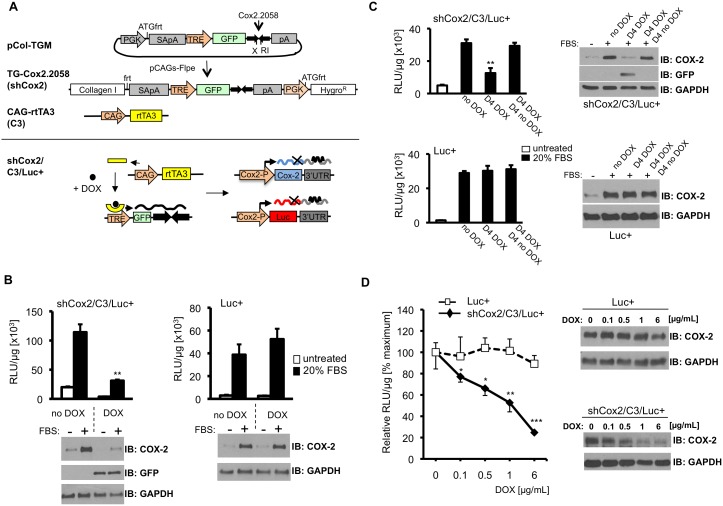
Inducible COX-2 shRNA expression suppresses *Cox2-*driven gene expression in cells cultured from triple transgenic mice. (**A**) Upper panel: The diagram shows the pCol-TGM targeting construct encoding GFP and COX-2 shRNA, expressed from a TRE promoter. Co-electroporation, with pCAGS-Flpe recombinase, into KH2 ES cells results in integration of the construct into the *ColA1* locus. ShCox2 mice are crossed with tet-transactivator mice (CAG-rtTA3/C3) to create double trangenics. Lower panel; in triple transgenic shCox2/C3/Luc+ mice, DOX-rtTA3 activates the TRE promoter, driving GFP and shCox2 expression; shCox2 blocks COX-2 and luciferase expression. Without DOX the TRE promoter is quiescent; COX-2 and luciferase are expressed normally. (**B**) Inducible suppression of *Cox2* gene expression in fibroblasts. Skin fibroblasts from triple transgenic shCox2/C3/Luc+ and Luc+ mice were cultured for four days in the absence or presence of DOX (1 µg/mL), shifted to 1% serum overnight, then stimulated with 20% serum for 6 hours. Luciferase activity was measured in extracts, COX-2 and GFP protein were analyzed by Western blot. Luciferase activity was normalized to protein content. (**C**) Reversible suppression of *Cox2* gene expression in skin fibroblasts. Cells were untreated (no DOX), treated for 4 days with DOX (D4 DOX), or treated for 4 days with DOX followed by 4 days without DOX (D4 DOX, D4 no DOX). Cells were stimulated with medium containing 20% FBS; luciferase activity, COX-2 expression and GFP expression were analyzed. (**D**) Bone marrow macrophages from shCox2/C3/Luc+ and control Luc+ mice were cultured in the indicated concentrations of DOX overnight, then stimulated for 4 hours with LPS (50 ng/mL) to induce COX-2. Cell extracts were analyzed for luciferase activity and COX-2 protein. Luciferase activities are normalized to LPS stimulation in the absence of DOX. Data are means +/− SD. Statistics compare DOX-treated cultures with cells not receiving DOX (*, p<0.05; **, p<0.01; ***, p<0.001).

To develop a single-copy transgenic mouse in which Cox2.2058 can be expressed in an inducible and reversible manner, we subcloned the Cox2.2058 sequence into the targeting vector (pCol-TGM, [24]) (Fig. 3A, upper panel). pCol-TGM contains a miR30-based expression cassette regulated by an inducible tetracycline response element (TRE) promoter. The pCol-TGM vector also contains, between the TRE promoter and the shRNA cloning site, a GFP coding region, which both enhances knockdown of target genes and enables easy tracking and isolation of shRNA-expressing cells [24]. Transgenic mice were generated using the Flp/FRT recombinase mediated cassette exchange (RMCE) strategy [22] to target the Cox2.2058 containing pCol-TGM sequence into the frt-hygro-pA ‘homing’ cassette of KH2 ES cells [20], [23], [24]. Fig. 3A (upper panel) shows the targeting vector (pCol-TGM) and the integrated construct after homologous recombination to create the TG-Cox2.2058 transgene allele. After selection and confirmation of ES cell clones with correct shRNA sequence, transgenic mice were generated using tetraploid embryo complementation [25]–[27].

#### Triple transgenic mice containing the CAG-rtTA3 (C3) reverse transactivator, the TG-Cox2

**2058 shRNA (shCox2) and the** ***Cox2^tm2Luc^*** **allele (Luc+) knockin.** The TRE promoter in TG-Cox2.2058 (shCox2) transgenic mice is activated only in the presence of a tetracycline-regulatable transactivator. We crossed the shCox2 mice to the CAG-rtTA3 (C3) reverse transactivator mouse line. The CAG promoter is particularly well expressed in skin epidermis [24]. Moreover, quantification of luciferase expression *in vivo* by non-invasive bioluminescent imaging is most sensitive and efficient in skin [36]. Consequently, to analyze the ability of shCox2 to reversibly suppress COX-2 expression we crossed *Cox2^tm2Luc/+^* (Luc+) reporter mice to the shCox2/C3 double transgenic mouse line. Triple transgenic mice carrying the CAG-rtTA3 transactivator (C3), the Cox2.2058 shRNA transgene (shCox2) and the *Cox2^tm2Luc^* allele (Luc+) develop normally. In subsequent experiments their luciferase expression is compared with single transgene heterozygous *Cox2^tm2Luc/+^* (Luc+) littermate controls.

#### Tet-regulated COX-2-shRNA reversibly suppresses expression from the *Cox2* gene in cultured cells

CAG-rtTA3 is a ‘tet-on’ transactivator, inactive unless a tetracycline analog such as doxycycline (DOX) is present. In the presence of DOX, rtTA3 should activate the TRE promoter, leading to shCox2 expression and subsequent down-regulation of *Cox2* transcripts that contain the targeted *Cox2* gene 3′UTR sequence (Fig. 3A lower panel). DOX withdrawal should inactivate the rtTA3 transactivator, and TRE-mediated transcription should cease.

CAG-rtTa3 driven gene expression is high in mouse skin [24]. We established primary skin fibroblast cultures both from triple transgenic shCox2/C3/Luc+ mice and from Luc+ control mice, to test DOX-regulated *Cox2* gene silencing. To examine whether DOX-induced shCox2 expression results in reduced expression from the *Cox2* gene locus, primary shCox2/C3/Luc+ skin fibroblasts and fibroblasts from Luc+ mice, cultured either in the presence or absence of DOX, were stimulated with 20% FBS, and assayed for luciferase activity. Skin fibroblasts from both mouse strains cultured in the absence of DOX demonstrate a substantial increase in FBS-stimulated luciferase activity in cell extracts over unstimulated cells (Fig. 3B). DOX reduced luciferase production in FBS-treated triple transgenic fibroblasts by ∼75% (Fig. 3B, left panel). COX-2 induction in FBS-stimulated triple transgenic fibroblasts was also substantially reduced in the presence of DOX (Fig. 3B, left panel). GFP expression in triple transgenic fibroblasts was observed only in DOX-treated cells, indicating that shCox2 production was DOX-dependent (Fig. 3B, Western blot). In contrast to results for fibroblasts from the triple transgenic cells, both luciferase activity and COX-2 protein expression in Luc+ cells were unaffected by DOX, ruling out non-specific side effects by DOX treatment (Fig. 3B, right panels).

To test the reversibility of DOX-dependent, shCox2-regulated COX-2 knockdown, fibroblasts from triple transgenic shCox2/C3/Luc+ and Luc+ mice were divided into three treatment groups. Cells were either treated with DOX for four days (D4 DOX), treated with DOX for 4 days followed by 4 days without DOX (D4 DOX, D4 no DOX) or never treated with DOX (no DOX). Cells were then treated with 20% FBS. DOX treatment (D4 DOX) resulted in significant reduction of luciferase and COX-2 expression in response to FBS stimulation in cells from triple transgenic (shCox2/C3/Luc+) mice, but not in FBS-stimulated cells from Luc+ mice (Fig. 3C). Moreover, FBS-stimulated luciferase activity and COX-2 protein expression following DOX withdrawal (D4 DOX, D4 no DOX), were identical to that observed in cells that were never exposed to DOX (no DOX), demonstrating that the DOX-regulated, shCox2-dependent inhibition of COX-2 expression and luciferase activity were fully reversible. GFP expression correlated strongly, in reciprocal fashion, with *Cox2* gene knockdown (Fig. 3C, upper right panel), confirming its role as a reliable biomarker of shCox2 expression.

Luciferase and COX-2 were strongly induced by LPS in primary bone marrow macrophages from both triple transgenic and Luc+ mice (Fig. 3D). Macrophages from both mouse strains were cultured overnight with increasing DOX concentrations, then stimulated with LPS. We observed dose-dependent reductions in luciferase activity and COX-2 expression in shCox2/C3/Luc+ macrophages, but not in Luc+ macrophages. These experiments demonstrate that co-expression of CAG-rtTA3 (C3) and TG-Cox2.2058 (shCox2) results in DOX-dependent, reversible inhibition of *Cox2* gene expression.

#### Doxycycline-regulated shCox2 expression suppresses *Cox2* gene expression in zymosan-induced inflammation in the mouse paw

Zymosan-induced paw inflammation in rodents is a classic localized inflammation model [37], [38]. We previously demonstrated that zymosan inflammation is accompanied by a robust, self-resolving COX-2 induction [28] that can be measured by repeated non-invasive optical imaging in the *Cox2^fLuc/+^*mouse [28]. Consequently, the paw inflammation model should also be very useful for initial characterization of the triple transgenic shCox2/C3/Luc+ mouse.

**Figure 4:**
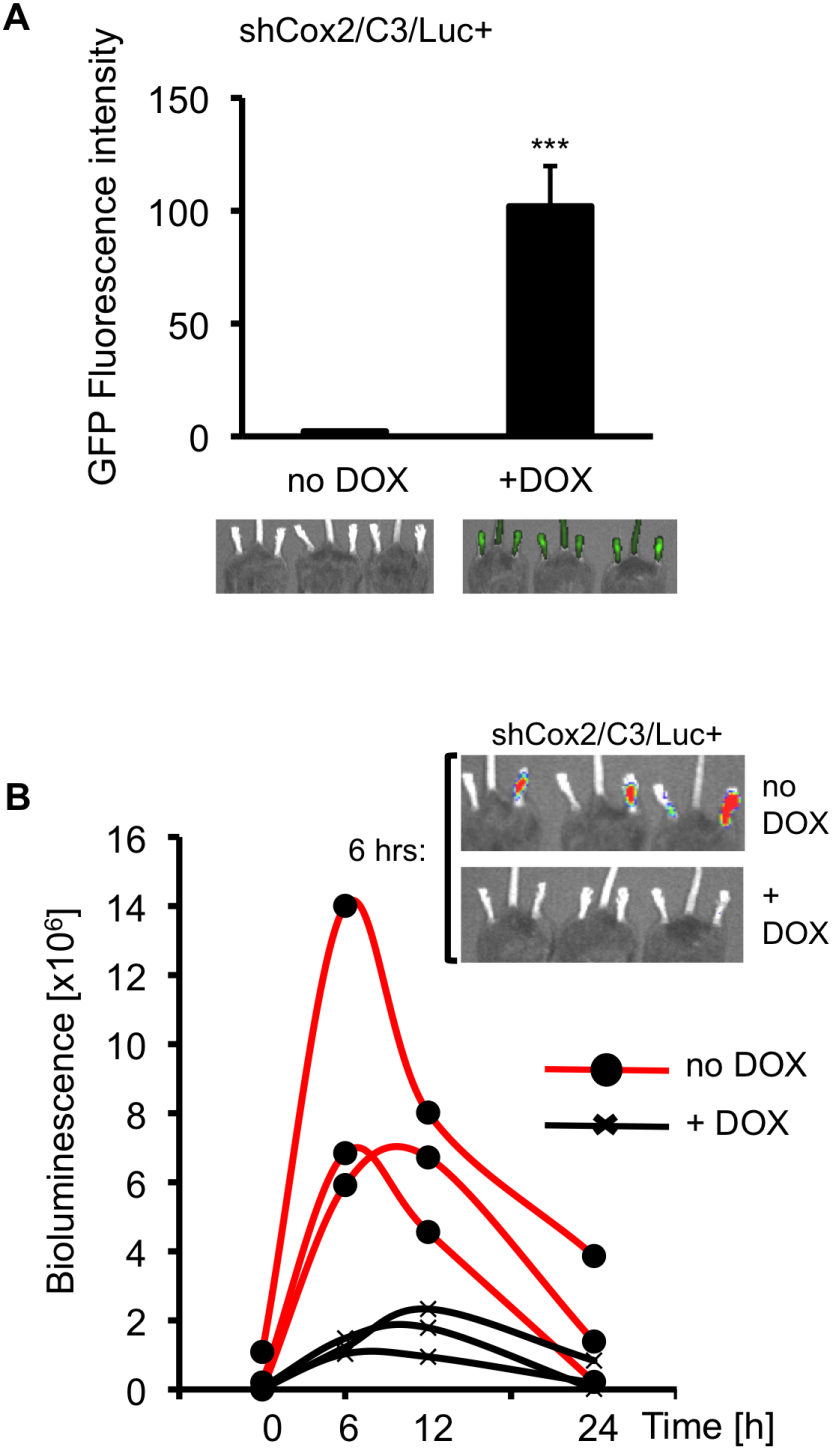
DOX-dependent suppression of *Cox2* driven luciferase expression in triple transgenic mice during zymosan-induced paw inflammation. (**A**) Fluorescent *in vivo* GFP imaging of shCox2/C3/Luc+ triple transgenic mice fed either a control diet (no DOX) or a DOX-containing diet (+DOX) for 12 days. GFP fluorescence was quantified with Living Image software. Data are means +/− SD, (n  = 3; ***, p<0.001). (**B**) ShCox2/C3/Luc+ mice were injected intraplantarly in the left hind paw with zymosan (30 µL, 2% w/v) and with saline in the contralateral hind paw. *In vivo* luciferase bioluminescence was non-invasively and repeatedly measured and quantified at the indicated time points. Each curve represents data from an individual animal. To control for individual animal variability the bioluminescence in the contralateral control saline-infected paw was subtracted from the bioluminescence value of the zymosan-injected paw for each observation.

To determine the dynamics of DOX-induced, shCox2-mediated, longitudinal silencing of the *Cox2* gene, three triple transgenic shCox2/C3/Luc+ mice received a DOX-containing diet for 12 days, while three additional triple transgenic mice were fed a control DOX-free diet. To analyze whether the shCox2 shRNA was expressed, and to obtain luciferase activity levels at baseline, the mice were imaged non-invasively for GFP fluorescence and for luciferase-dependent bioluminescent activity prior to zymosan administration (Fig. 4A and 4B, 0 time point). GFP fluorescence from the paws of mice that received the DOX-containing diet (+DOX) was greater than 10-fold increased when compared to paws of mice that did not receive DOX, indicating that shCox2 expression was strongly induced by DOX (Fig. 4A).

A 2% zymosan suspension in saline (30 µL) was then injected sub-plantar into the left rear paws of all six shCox2/C3/Luc+ mice, and saline was injected into the right rear (contralateral) paws. Luciferase expression from the *Cox2^tm2Luc^* allele was measured by bioluminescence imaging at 6, 12, and 24 hours post zymosan injection (Fig. 4B). Zymosan injection resulted in rapid luciferase accumulation compared to luciferase activity at baseline, prior to zymosan injections; zymosan-induced luciferase activity peaked between 6 and 12 hours, then decreased to near-baseline values within 24 hours (Fig. 4B). *Cox2* gene-directed luciferase activity was significantly higher throughout the induction period in mice that received a DOX-free diet (no DOX) compared with mice fed a DOX-containing diet. Thus DOX pretreatment resulted in significant inhibition of *Cox2* gene-driven luciferase expression during zymosan-induced paw inflammation.

Zymosan-induced luciferase activity in Luc+ control mice fed a DOX-containing diet was not significantly different from luciferase activity observed in shCox2/C3/Luc+ mice on a DOX-free diet, confirming that the reduction in luciferase activity observed in shCox2/C3/Luc+ mice on DOX was not simply due to the presence of DOX (Fig. S3A).

#### Doxycycline-regulated shCox2 reversibly suppresses *Cox2* gene expression in TPA-treated mouse skin

Topical TPA administration induces an inflammatory reaction in mouse skin [39]–[42]. We used repeated TPA skin treatment of shCox2/C3/Luc+ triple transgenic mice, in combination with repeated noninvasive imaging, to evaluate reversible inactivation of *Cox2*-driven luciferase expression by DOX-inducible shCox2 expression in individual mice.

**Figure 5:**
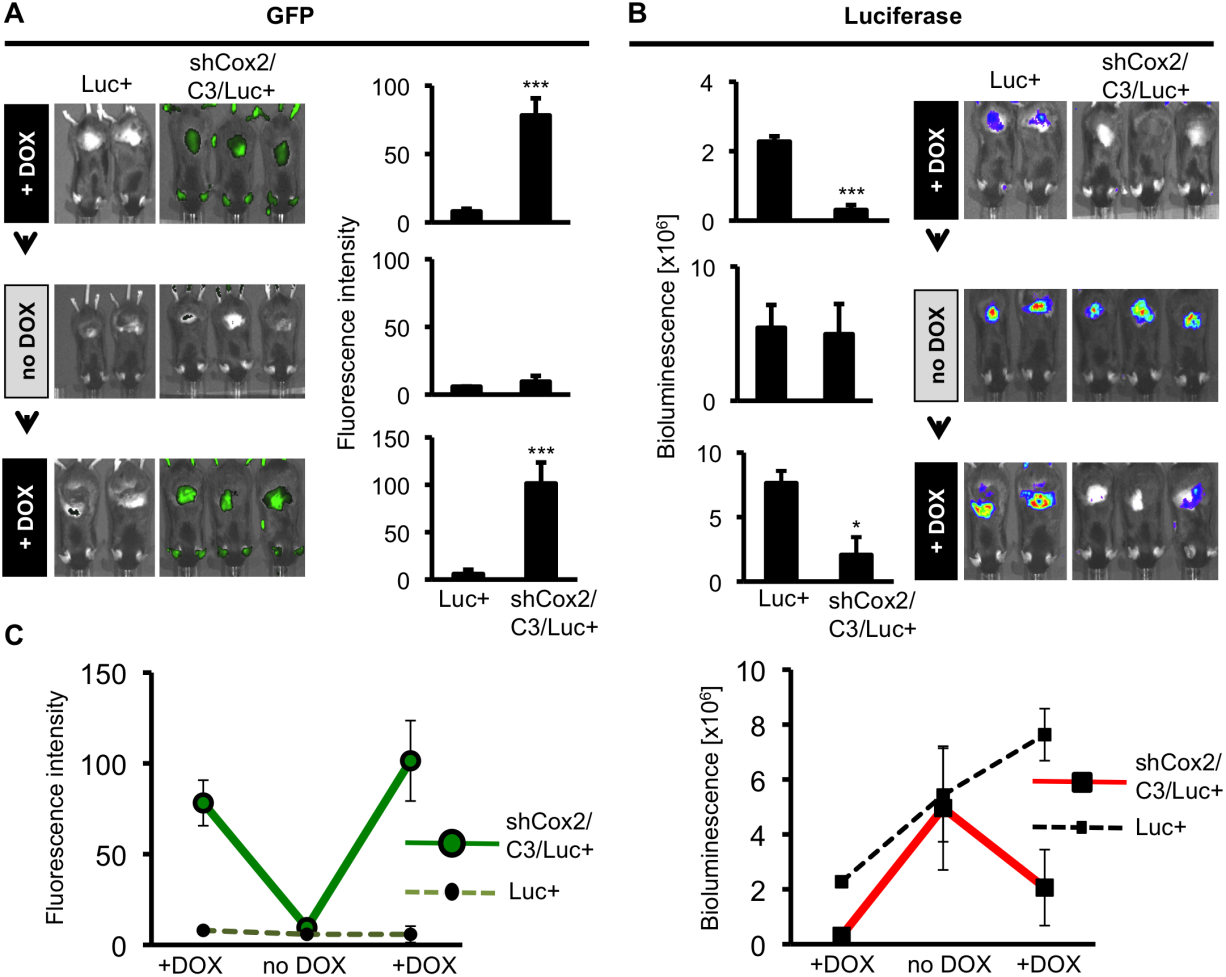
Reversible DOX-dependent suppression of *Cox2-*driven luciferase expression in skin of TPA-treated triple transgenic mice. Luc+ mice and triple transgenic mice were subjected to the following diet, TPA skin application and imaging schedule: Mice were placed on a DOX-supplemented diet (+DOX) for 12 days followed by skin TPA application. Mice were imaged 24 hours later for GFP fluorescence and luciferase bioluminescence. The mice were then shifted to a DOX-free diet (no DOX) for 12 days and the skin TPA application, GFP fluorescence and luciferase bioluminescence analyses were repeated a second time. The mice were then shifted back to a DOX-supplemented diet (+DOX) for 12 days and the skin TPA application, GFP fluorescence and luciferase bioluminescence analyses were repeated a third time. (**A**) GFP fluorescence, indicating shCox2 expression. Data are means +/− S.D. (***p<0.001). After the DOX-diet was removed (middle panel, no DOX), GFP fluorescence returned to baseline (p>0.05, ns). (**B**) Luciferase bioluminescence, indicating *Cox2* gene-driven luciferase expression. TPA-induced luciferase expression is reversibly reduced, in the presence of DOX, in triple transgenic mice. Data are means +/− S.D (*p<0.05, ***p<0.001). (**C**) Summary of GFP fluorescent intensity values (left panel) and bioluminescence (right panel) in Luc+ mice (dashed lines) and shCox2/C3/Luc+ mice (solid lines) for the three successive non-invasive imaging analyses. Error bars show S.D.

Three triple transgenic shCox2/C3/Luc+ mice and two control Luc+ littermate control mice received a DOX-containing diet for 12 days. A small area on the back of the mice was shaved and expression from the *Cox2* gene was induced by applying TPA. Twenty-four hours later GFP, as a measure of shCox2 expression, and luciferase activity were measured by respective non-invasive optical fluorescent and bioluminescent imaging (Figs. 5A and B, top rows). After imaging, the DOX diet was replaced with a DOX-free diet. Twelve days later mice were again subjected to TPA treatment on their back and again imaged 24 hours later for GFP and luciferase expression (Figs. 5A and B, middle rows). After imaging, the mice were switched back to a DOX-containing diet for 12 days, treated again with TPA, and imaged a third time for DOX-mediated GFP and *Cox2* gene-mediated luciferase expression (Fig. 5A and B, third rows) to determine whether *Cox2* shRNA-mediated knockdown of *Cox2* gene-driven transcripts is reversible.

DOX induced a ∼10-fold GFP fluorescence induction in skin of triple transgenic shCox2/C3/Luc+ mice when compared to fluorescence from Luc+ control mice, which do not contain the GFP-linked shRNA transgene (Fig. 5A, +DOX, top row). Twelve days after the DOX diet was replaced with a DOX-free diet, skin GFP expression returned to baseline (Fig. 5A, middle row), suggesting that the COX-2-shRNA is no longer expressed. When shCox2/C3/Luc+ mice were returned to a DOX-containing diet for 12 days, they again showed a ∼10-fold induction of GFP expression (Fig. 5A, bottom row). These data demonstrate that shCox2 expression can be reversibly up-and-down-regulated within the same mouse, in response to the presence or absence of DOX in the diet.

In the presence of DOX in the diet, when DOX-dependent TG-Cox22058 (shCox2) is strongly expressed (Fig. 5A, top row), TPA elicited little or no *Cox2* gene-driven luciferase expression in triple transgenic shCox2/C3/Luc+ mice (Fig. 5B, top row). In contrast, TPA painting of Luc+ mice, which do not contain the shCox2 transgene, elicits robust luciferase expression. Luciferase bioluminescence in shCox2/C3/Luc+ mice was reduced by ∼80% relative to luciferase expression in Luc+ mice when the mice are on a DOX-containing diet (Fig. 5B, top row). However, when DOX is removed from the diet of shCox2/C3/Luc+ mice, a condition under which there is no expression of DOX-dependent shCox2, indicated by a lack of GFP expression (Fig. 5A, middle row), TPA induced equivalent levels of *Cox2* gene-dependent luciferase expression in shCox2/C3/Luc+ mice and the control Luc+ mice (Fig. 5B, middle row). When the shCox2/C3/Luc+ mice are returned to a DOX-containing diet (when DOX-dependent shCox2 is once again expressed; Fig. 5A, bottom row), TPA-induced luciferase expression is once again reduced in shCox2/C3/Luc+ mice (by 75%) relative to luciferase expression in Luc+ mice (Fig. 5B, bottom row). Luc+ mice on DOX-containing and DOX-free diets expressed identical luciferase activities in response to TPA treatment; DOX treatment alone did not influence luciferase induction by TPA in skin (Fig. S3B).

In Fig. 5C we plot the quantification of the successive GFP/shCox2 expression (left panel) and successive *Cox2* gene-driven luciferase expression (right panel) in shCox2/C3/Luc+ mice and control Luc+ mice. In the presence of DOX, GFP/shCox2 is strongly induced, both initially and again following a period of DOX withdrawal; however, DOX withdrawal reduces shCox2 expression in the triple transgenic mice to the same background level observed in Luc+ mice (Fig. 5C, left panel). The inverse is true for *Cox2* gene-driven luciferase in the triple transgenic shCox2/C3/Luc+ mice; in the presence of DOX, when shCox2 is expressed, luciferase expression is strongly inhibited; in the absence of shCox2 expression (in the absence of DOX) there is no inhibition of *Cox2* gene-driven luciferase expression relative to Luc+ mice. Repeated DOX administration once again suppresses TPA-induced luciferase induction in the shCox2/C3/Luc+ mice. The increasing luciferase activity observed in Luc+ mice with successive TPA treatments (Fig. 5C, right panel, dotted line) reflects the hyperalgesic response observed for COX-2 expression with repeated inflammatory insult [43]–[46]. In summary, these results show that shCox2 expression can be reversibly regulated *in vivo* by DOX and that reversible regulation of shCox2 expression results in reciprocal downregulation of *Cox2* transcripts.

#### Doxycycline-regulated shCox2 expression can suppress induction of COX-2 protein and leukocyte infiltration in mice homozygous for the wild-type *Cox2* gene

Heterozygous *Cox2^tm2Luc/+^* mice, expressing luciferase from one allele and COX-2 protein from the other allele, are very useful in assessing reversible and/or prolonged *Cox2* gene silencing by repeated non-invasive imaging of the same animal. However, to employ this system to study the reversible, cell-specific role of COX-2 in biological contexts, it is essential to demonstrate that the single copy of the COX-2 shRNA present in double transgenic TG-Cox2.2058/CAG-rtTA3 (shCox2/C3) mice can, when responding to DOX, suppress COX-2 expression in mice with two functional *Cox2* alleles.

**Figure 6:**
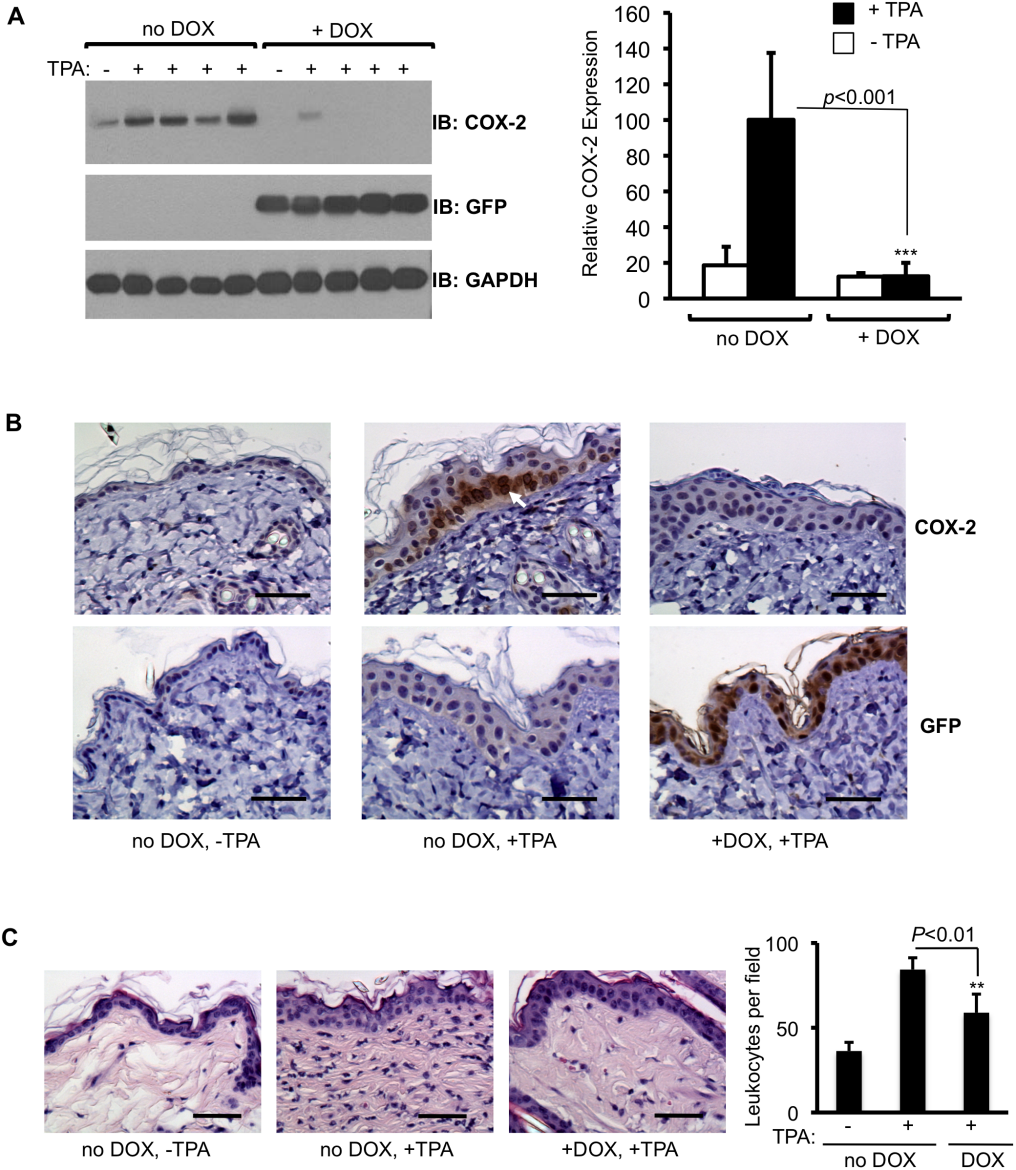
DOX-dependent suppression of TPA-induced COX-2 expression by shCox2 in mice homozygous for the *Cox2* gene. (**A**) Double transgenic shCox2/C3 mice, which have two wild type *Cox2* alleles, were maintained on either a control (no DOX) or a DOX-supplemented diet (+DOX) for 12 days, then painted with TPA on the back. 24 hours after TPA administration the mice were euthanized and skin extracts were assayed for GFP and COX-2 protein by Western blotting. Quantification is from three independent experiments. The COX-2 signal was normalized to the GAPDH loading control. Data are means +/− S.D. (***p<0.001). (**B**) COX-2 (upper panels) and GFP (lower panels) immunohistochemistry in skin of untreated shCox2/C3 mice (left panels), 24 hours after TPA administration to shCox2/C3 mice maintained on a DOX-free diet (center panels) and 24 hours after TPA treatment of shCox2/C3 mice maintained for 12 days on a DOX-supplemented diet (right panels). COX-2 staining is visible as brown patches (white arrow) in the basal epithelium. (**C**) H&E stain to visualize leukocytes in skin sections from double transgenic shCox2/C3 mice treated with TPA and/or DOX as in B. The graph depicts quantification of leukocytes in the dermis. Quantification is from two independent experiments. Data are means +/− S.D. (**p<0.01). The scale bar indicates 50 µm.

TPA treatment induced COX-2 expression in the skin of double transgenic shCox2/C3 mice on a DOX-free diet (Fig. 6A, left panel). In contrast, COX-2 expression was not induced above baseline in TPA-treated double transgenic mice on the DOX-supplemented diet, in which shCox2 was expressed (Fig. 6A, left panel). Quantification of Western blot signals from three independent experiments showed that the average knockdown of COX-2 protein accumulation in the skin of DOX-treated mice following TPA treatment was ∼80–90%, corresponding to near baseline expression (Fig. 6A, right panel).

To analyze where in the skin COX-2 expression occurs in response to TPA treatment and to visualize the consequences of DOX-induced shCox2 expression, COX-2 and GFP expression in TPA-treated shCox2/C3 double transgenic mice were analyzed by immunohistochemistry (Fig. 6B). TPA-induced COX-2 expression was visible predominantly in the basal layer of the skin epithelium in mice on a DOX-free diet (Fig. 6B, white arrow). In contrast, no COX-2 staining could be observed in the skin of TPA-treated shCox2/C3 mice either on a DOX-supplemented diet or in untreated control mice (Fig. 6B). GFP staining reflecting shCox2 expression was visible throughout the epithelial layer in mice that received a DOX-containing diet, but was not present in mice on a DOX-free diet (Fig. 6B, lower panel). These observations confirm the Western blot data, demonstrating that single copy shCox2 expression from the DOX-activated TRE locus is sufficient to suppress TPA-induced COX-2 expression in skin of mice homozygous for the wild-type *Cox2* gene.

Our final experiment was to demonstrate inhibition of a COX-2 dependent phenotypic function as a consequence of RNA interference. TPA-treatment of mouse skin results in a COX-2 dependent increase in leukocyte infiltration [39]. To determine whether shCox2-mediated suppression of TPA-induced COX-2 expression in skin results in functional COX-2 attenuation, we analyzed the consequences of DOX treatment on leukocyte infiltration following TPA stimulation in skin of double transgenic shCox2/C3 mice. On a DOX-free diet, we observed an increased number of leukocytes in the dermis of mice treated with TPA compared to untreated control mice (36.3+/−5.1 vs. 84.3+/−6.9 cells per field) (Fig. 6C). In contrast, on a DOX-supplemented diet, TPA-induced leukocyte infiltration was significantly reduced in the dermis of TPA-treated shCox2/C3 mice compared to TPA-treated mice on a control diet (58.9+/−10.9 vs. 84.3+/−6.9) (Fig. 6C).

In summary, we have established a mouse strain that expresses a tet-regulatable, miR30-based shRNA targeting the *Cox2* transcript, and have demonstrated reversible and functional DOX-mediated suppression of *Cox2* gene expression.

## Discussion

In this report we describe the creation of a mouse model in which COX-2 expression can be cell-specifically and reversibly regulated by a doxycycline-inducible shRNA that targets the *Cox2* transcript. In addition, we created a luciferase reporter knockin mouse (*Cox2^tm2Luc^*), in which luciferase replaces the coding region of the endogenous *Cox2* gene, while retaining all *Cox2* regulatory regions (promoter, 5′UTR, 3′UTR). Because shRNA Cox2.2058 targets a sequence in the *Cox2* gene 3′UTR, *Cox2* gene-driven luciferase activity can be used to monitor noninvasively Cox2.2058 shRNA-mediated COX-2 knockdown. Using the *Cox2^tm2Luc/+^* heterozygous reporter mouse, investigators can quickly evaluate the feasibility of the system for tissue or cell-specific applications.

Although the heterozygous *Cox2^tm2Luc/+^* mouse provides a model in which to quickly monitor *Cox2* gene expression, many aspects of COX-2 biology are gene-dosage dependent [28], [47], [48]. Since the TG-Cox2.2058 transgene is present in only a single copy, it is necessary to demonstrate substantial DOX-dependent COX-2 suppression in mice homozygous for the *Cox2* wild type allele and, more importantly, to confirm that COX-2 knockdown can result in functional suppression. Both criteria are met for TPA-induced skin inflammation (Fig. 6), a classic model of COX-2 induction [49]. ShCox2 silencing of COX-2 expression resulted in a significant reduction of leukocytes present in the dermis following TPA treatment (Fig. 6C). This finding is consistent with previous studies that show that COX-2 plays a role in leukocyte chemotaxis [39], [50]; Nakamura et al [39] found a ∼40% reduction in leukocyte infiltration following two TPA applications in the presence of the COX-2 inhibitor nimesulide [39].

In our *in vivo* experiments (Figs. 4–6) we supply and withdraw DOX in 12-day intervals. The literature suggests that DOX induction and de-induction occurs more rapidly; e.g. 2–4 days [24]. However, when monitoring GFP as a surrogate indicator for shCox2 expression, we observed residual fluorescence for 10–12 days after DOX withdrawal. Additional experiments will be needed to determine the relationship between the stability of the GFP reporter and the stability of Cox2.2058 following DOX removal, to optimize minimal times required for reversible, Cox2.2058-mediated COX-2 expression knockdown and recovery.

Measuring *Cox2-*driven luciferase activity confirmed that DOX-regulated shCox2-dependent COX-2 silencing exhibited full reversibility in the skin inflammation system (Fig. 5). The ability to reversibly and repeatedly knock down *Cox2* gene expression is one of the greatest assets of the shCox2 gene silencing protocol; the model has the enormous advantage of not permanently disrupting the *Cox2* gene. This property allows reversible manipulation of COX-2 expression at any time after disease onset, permitting – for example – investigation of the complex roles of COX-2 expression in alternative cells in inflammatory responses to pathogens, by initiating knockdown in specific cell types at different time points after infection. In contrast, conventional *Cox2* gene disruption permanently eliminates global COX-2 expression at all times.

Although conditional Cre recombinase expression can also be induced at distinct time points by using the inducible Cre-ERT/tamoxifen system, recombination irreversibly disrupts the target locus. Moreover, Cre expression can, in some instances, show significant toxicity [51]. In addition, shCox2 gene silencing requires only two genes; a single TG-Cox2.2058 transgene and the appropriate rtTA or tTA transactivator transgene; Cre-based conditional deletion requires three alleles (two *Cox2^fl^* ^o*x*^ alleles and the targeted Cre recombinase); consequently, shCox2 targeting can be more easily and rapidly combined with existing mouse disease models. The combination of easier genetic modeling and repeated, reversible gene silencing should permit insights that go beyond what can be obtained through conditional knockout approaches. Another advantage of inducible shRNA gene silencing which distinguishes it from the binary Cre-loxP system is the possibility of graded inhibition of gene expression by varying DOX dosage.

Suppression by shCox2 in distinct cell types requires appropriate cell/tissue-specific rtTA/tTA transgenic mice. The list of such mice is already extensive (ww.tetsystems.com), and growing. In addition, a CAG promoter-driven lox-stop-lox conditional rtTA transactivator that enables use of a Cre driver to convert transgenic mice to a cell-specific rtTA setting is currently under development (S. Lowe, submitted for publication).

Despite their great utility, RNAi-based gene regulation systems have their limitations. Tetracycline analogues can have side effects [52]. We included Luc+ control animals, to verify that COX-2 inhibition was due to shRNA expression, and not to DOX side effects (Fig. S3). It is also important to ensure that DOX efficiently reaches the target tissue of interest; DOX in the diet is more effective than DOX supplied in the drinking water [53].

Adequate rtTA expression in the target tissue/cell type is a key, rate-limiting step for DOX-regulated gene silencing. We chose the CAG-rtTA3 transactivator, which shows particularly strong expression in skin [24], to facilitate initial testing of the Cox2.2058 shRNA. The CAG promoter is considered by many to be ubiquitous. Because of our interest in inflammatory roles of COX-2, we also analyzed luciferase activity in spleens and lungs of IFN-γ + LPS-treated triple transgenic mice. Luciferase expression in the spleen or lung was not reduced significantly in IFN-γ + LPS treated mice that received a DOX-supplemented diet (Fig. S4). Analysis of DOX-dependent shRNA expression in target cells is facilitated by GFP co-expression. Consistent with previous reports [24], we observed less than two-fold shRNA-coupled GFP induction in spleen and lung by DOX, in contrast to the greater than 10-fold GFP induction in skin. These results suggest that CAG-driven rtTA3 transactivator expression may not be sufficient to block COX-2 expression in all tissues, or that silencing of the TRE-driven transgene may occur in some tissues, emphasizing the importance of systematic validation of the system for each application. Finally, it is important to keep in mind that, although recent advances in design of the microRNA backbone have significantly increased the effectiveness of single copy RNAi molecules [54], tet-regulated RNAi can rarely produce the null phenotype in individual cells achievable by gene deletion strategies.

Inducible *Cox2* gene knockdown using the tet-regulated shCox2 expression system provides a complementary approach to global and conditional *Cox2* gene knockout strategies to study COX-2 roles *in vivo*. Because COX-2 is inducible by a wide range of stimuli and plays an important role in many disease states, the double transgenic shCox2/C3 mouse and the triple transgenic shCox2/C3/Luc+ mouse should become useful tools for the study of the many aspects of COX-2 biology.

## Supporting Information

Figure S1 **(A) Percent of GFP expressing NIH 3T3 cells, two days after LMP retrovirus vector transduction at a low multiplicity of infection (MOI).** Less than 1% of the cells are GFP positive. The cells were harvested, washed with PBS, fixed with 0.01% paraformaldehyde and analyzed by flow cytometry to determine the percentage of GFP expression. Mock; untransduced cells, indicating background fluorescence. Control; cells transduced with a LMP vector encoding a control shRNA against luciferase. Cox2.284 (1) – Cox2.3711 (4); cells transduced with LMP vectors encoding *Cox2* specific shRNAs 1–4. **(B)** Schematic representation of the LMP retrovirus vector construct, indicating the probe used for Southern blot (red) and restriction enzyme target sites (X, *Xho*I. RI, *Eco*RI. HIII, *Hind*III. NI, *Nco*I). **(C–D)** Southern blot of LMP vector transduced NIH 3T3 cells. Cells were transduced at a low MOI. Two days later the cells were treated with 2.5 µg/mL puromycin to select for LMP transduced cells. Total DNA was harvested using the DNeasy kit (Qiagen). Cell DNA was digested with restriction enzymes overnight, before being subjected to Southern blot analysis. **(C)** Southern blot of *Hind*III digested DNA from mock transduced NIH 3T3 cells (m) or NIH 3T3 cells transduced with the LMP retrovirus vector encoding control (c) or *Cox2* specific shRNAs (1–4). *Hind*III digestion results in a 1100 bp fragment within the vector backbone, confirming the integrated LMP vector in cell genomic DNA. **(D)** Southern blot of *Nco*I digested DNA from mock-transduced (m), LMP control vector-transduced (c) or LMP vector encoding Cox2.5078 shRNA (3)-transduced NIH3T3 cells. *Nco*I has a single target site close to the 3′ end of the LMP vector, resulting in a ∼1000+ bp fragment depending on the location of the closest *Nco*I site in the cellular genome. The characteristic smear on the Southern blot confirms multiple-sized DNA fragments, indicating divers integration sites.
(TIF)

Figure S2 **COX-2 and luciferase induction by interferon gamma and endotoxin (IFN-γ/LPS) in the hearts, livers and lungs of heterozygous** ***Cox-2^tm2Luc/+^*** **mice.** Four mice were injected i.p. with IFNγ followed two hours later with LPS, or with saline. After 6 hours, mice were euthanized, tissues were removed and luciferase bioluminescence was quantified by bioluminescence imaging (left panels). Tissue extracts were then prepared and luciferase enzymatic activity was measured. COX-2 protein expression in tissue extracts was also analyzed by immunoblotting (right panels). Data are means +/− SD (**, p<0.01; *, p<0.05).
(TIF)

Figure S3 **(A) Luc+ mice that receive a DOX containing diet (indicated in blue) have similar luciferase expression in the zymosan treated paws at 6 hours compared to triple transgenic (shCox2/C3/Luc+) mice that do not receive a DOX containing diet (indicated in red), confirming that DOX treatment alone does not significantly reduce luciferase expression.** Data are means +/− SD, *p<0.05. **(B)** DOX in the diet does not affect TPA-induced *Cox2-*driven luciferase expression in skin. Luc+ mice were maintained either on a DOX-free diet or on a DOX-supplemented diet for 12 days prior to TPA administration to the skin. *In vivo* luciferase bioluminescence was measured 24 hours later. Bioluminescence in response to TPA painting is not significantly different in Luc+ control mice on DOX-free and DOX-supplemented diets. Data are individual values and averages from two mice.
(TIF)

Figure S4 **(A) GFP fluorescence imaging of spleen and lung tissue from triple transgenic shCox2/C3/Luc+ mice that received a DOX containing diet (+DOX) or a control diet (no DOX) for 12 days.** GFP fluorescence is increased significantly, but not to a great extent in the spleens. In the lungs, there was no significant difference in GFP fluorescence between shCox2/C3/Luc+ mice on a DOX containing diet and on a DOX free diet. **(B)** Luciferase induction by interferon gamma and endotoxin (IFN-γ/LPS) in the spleens and lungs of triple transgenic shCox2/C3/Luc+ mice that were fed a DOX containing diet (+DOX) to induce shRNA expression or a control diet (no DOX) for 12 days. Mice were injected i.p. with IFNγ followed two hours later with LPS, or with saline (mock). After 6 hours, mice were euthanized, tissues were removed and luciferase bioluminescence was quantified by bioluminescence imaging. Luciferase expression was not significantly different in mice receiving the DOX diet versus controls. Data are means +/− SD. (*, p<0.05, *ns,* p>0.05, n = 4).
(TIF)
