# Supplementary figures and images for: Reversible Suppression of Cyclooxygenase 2 (COX-2) Expression In Vivo by Inducible RNA Interference
Source: PLoS One. 2014 Jul 2;9(7):e101263. doi: 10.1371/journal.pone.0101263 (PMC4079684; doi:10.1371/journal.pone.0101263)

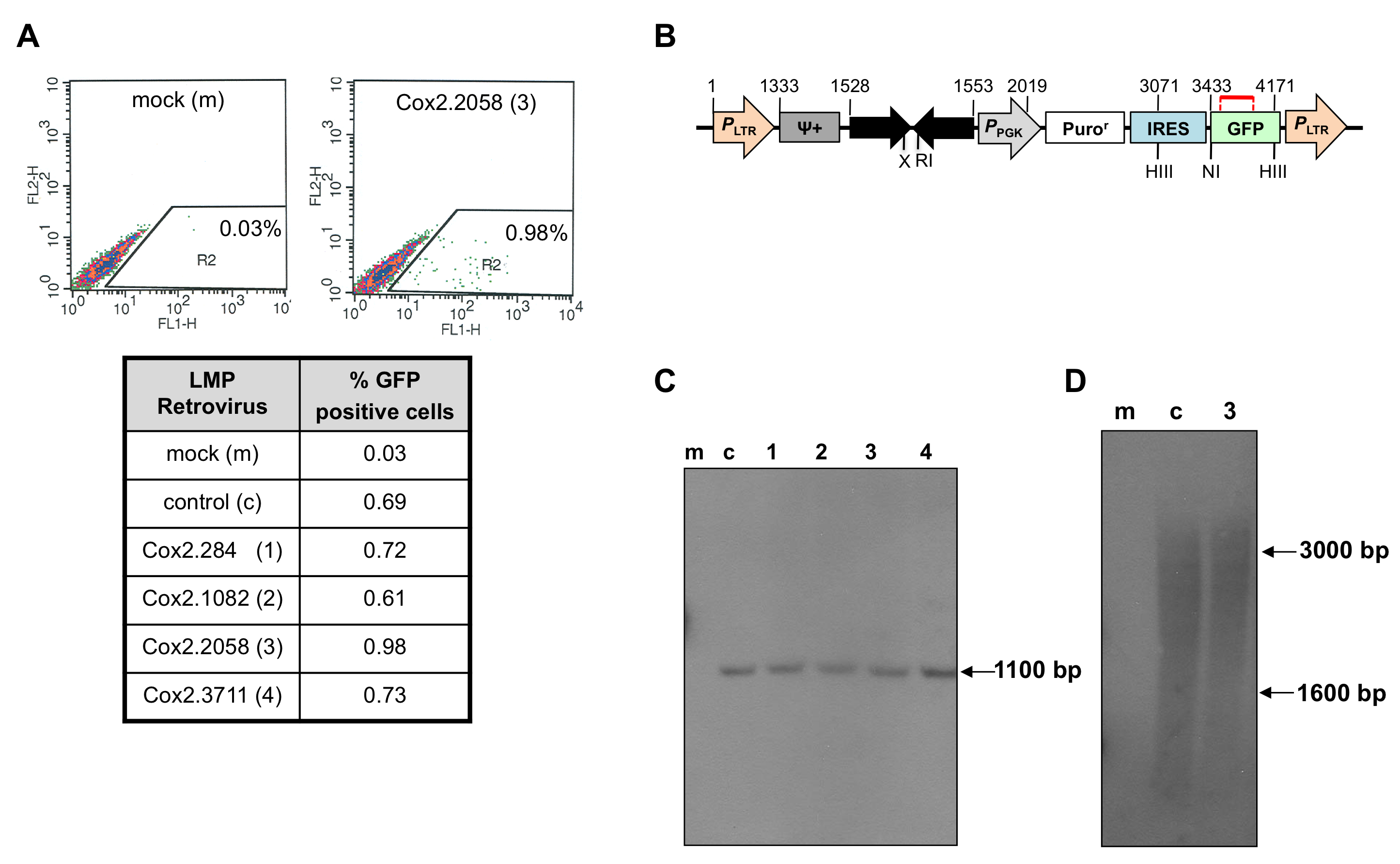

Supplement: Figure S1 — (A) Percent of GFP expressing NIH 3T3 cells, two days after LMP retrovirus vector transduction at a low multiplicity of infection (MOI). Less than 1% of the cells are GFP positive. The cells were harvested, washed with PBS, fixed with 0.01% paraformaldehyde and analyzed by flow cytometry to determine the percentage of GFP expression. Mock; untransduced cells, indicating background fluorescence. Control; cells transduced with a LMP vector encoding a control shRNA against luciferase. Cox2.284 (1) – Cox2.3711 (4); cells transduced with LMP vectors encoding Cox2 specific shRNAs 1–4. (B) Schematic representation of the LMP retrovirus vector construct, indicating the probe used for Southern blot (red) and restriction enzyme target sites (X, XhoI. RI, EcoRI. HIII, HindIII. NI, NcoI). (C–D) Southern blot of LMP vector transduced NIH 3T3 cells. Cells were transduced at a low MOI. Two days later the cells were treated with 2.5 µg/mL puromycin to select for LMP transduced cells. Total DNA was harvested using the DNeasy kit (Qiagen). Cell DNA was digested with restriction enzymes overnight, before being subjected to Southern blot analysis. (C) Southern blot of HindIII digested DNA from mock transduced NIH 3T3 cells (m) or NIH 3T3 cells transduced with the LMP retrovirus vector encoding control (c) or Cox2 specific shRNAs (1–4). HindIII digestion results in a 1100 bp fragment within the vector backbone, confirming the integrated LMP vector in cell genomic DNA. (D) Southern blot of NcoI digested DNA from mock-transduced (m), LMP control vector-transduced (c) or LMP vector encoding Cox2.5078 shRNA (3)-transduced NIH3T3 cells. NcoI has a single target site close to the 3′ end of the LMP vector, resulting in a ∼1000+ bp fragment depending on the location of the closest NcoI site in the cellular genome. The characteristic smear on the Southern blot confirms multiple-sized DNA fragments, indicating divers integration sites. (TIF) [file pone.0101263.s001.tif]

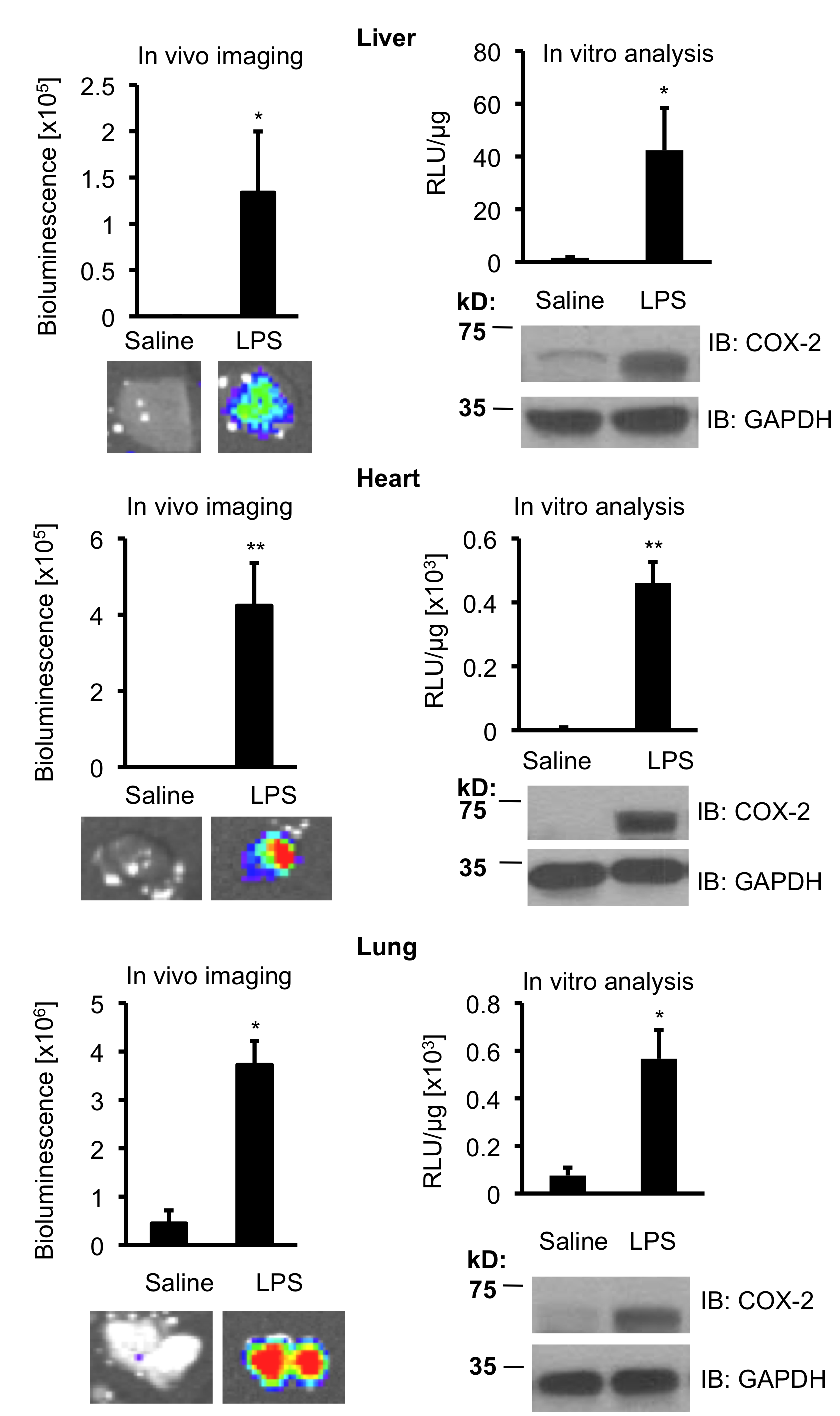

Supplement: Figure S2 — COX-2 and luciferase induction by interferon gamma and endotoxin (IFN-γ/LPS) in the hearts, livers and lungs of heterozygous Cox-2tm2Luc/+ mice. Four mice were injected i.p. with IFNγ followed two hours later with LPS, or with saline. After 6 hours, mice were euthanized, tissues were removed and luciferase bioluminescence was quantified by bioluminescence imaging (left panels). Tissue extracts were then prepared and luciferase enzymatic activity was measured. COX-2 protein expression in tissue extracts was also analyzed by immunoblotting (right panels). Data are means +/− SD (**, p<0.01; *, p<0.05). (TIF) [file pone.0101263.s002.tif]

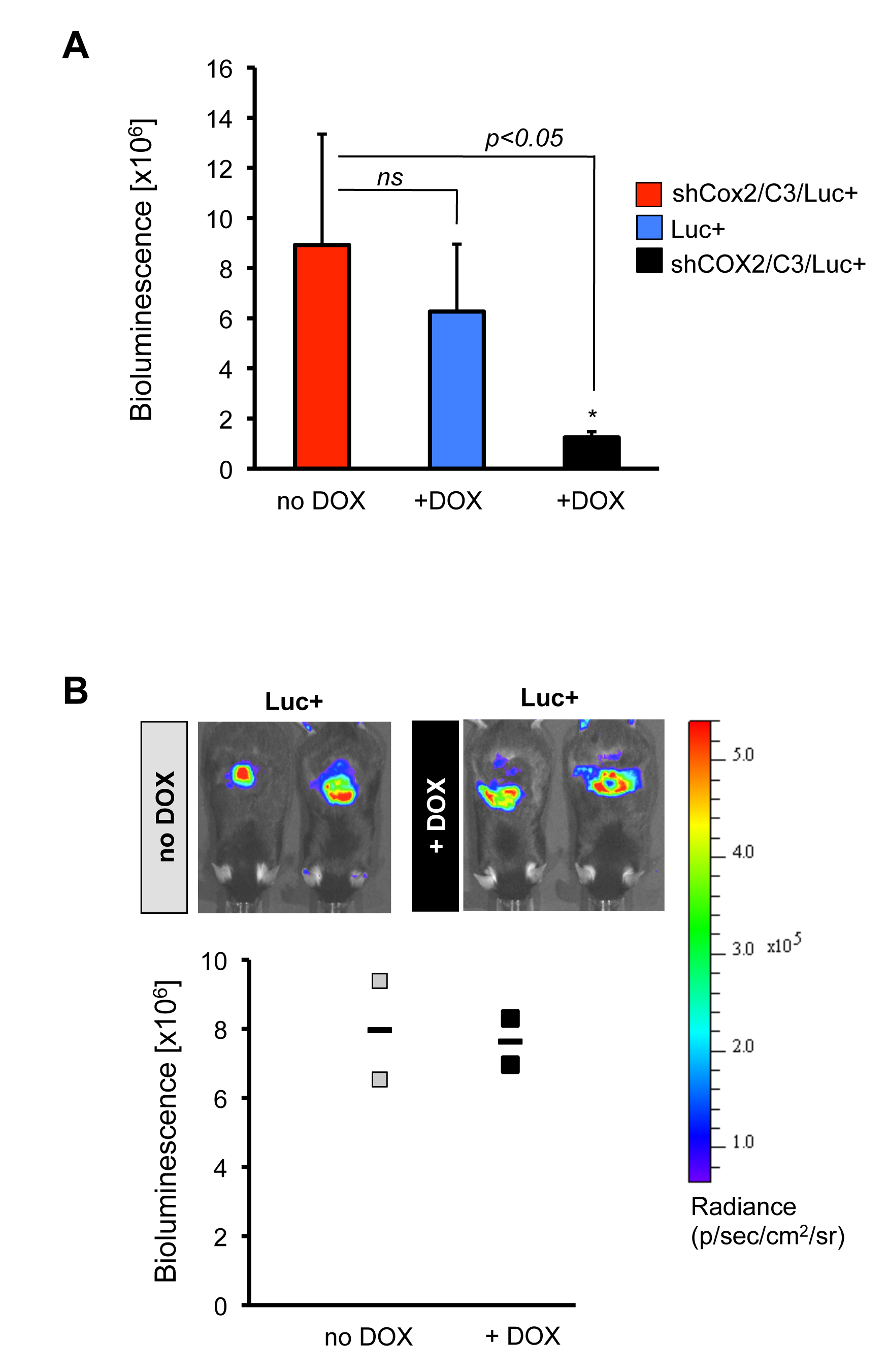

Supplement: Figure S3 — (A) Luc+ mice that receive a DOX containing diet (indicated in blue) have similar luciferase expression in the zymosan treated paws at 6 hours compared to triple transgenic (shCox2/C3/Luc+) mice that do not receive a DOX containing diet (indicated in red), confirming that DOX treatment alone does not significantly reduce luciferase expression. Data are means +/− SD, *p<0.05. (B) DOX in the diet does not affect TPA-induced Cox2-driven luciferase expression in skin. Luc+ mice were maintained either on a DOX-free diet or on a DOX-supplemented diet for 12 days prior to TPA administration to the skin. In vivo luciferase bioluminescence was measured 24 hours later. Bioluminescence in response to TPA painting is not significantly different in Luc+ control mice on DOX-free and DOX-supplemented diets. Data are individual values and averages from two mice. (TIF) [file pone.0101263.s003.tif]

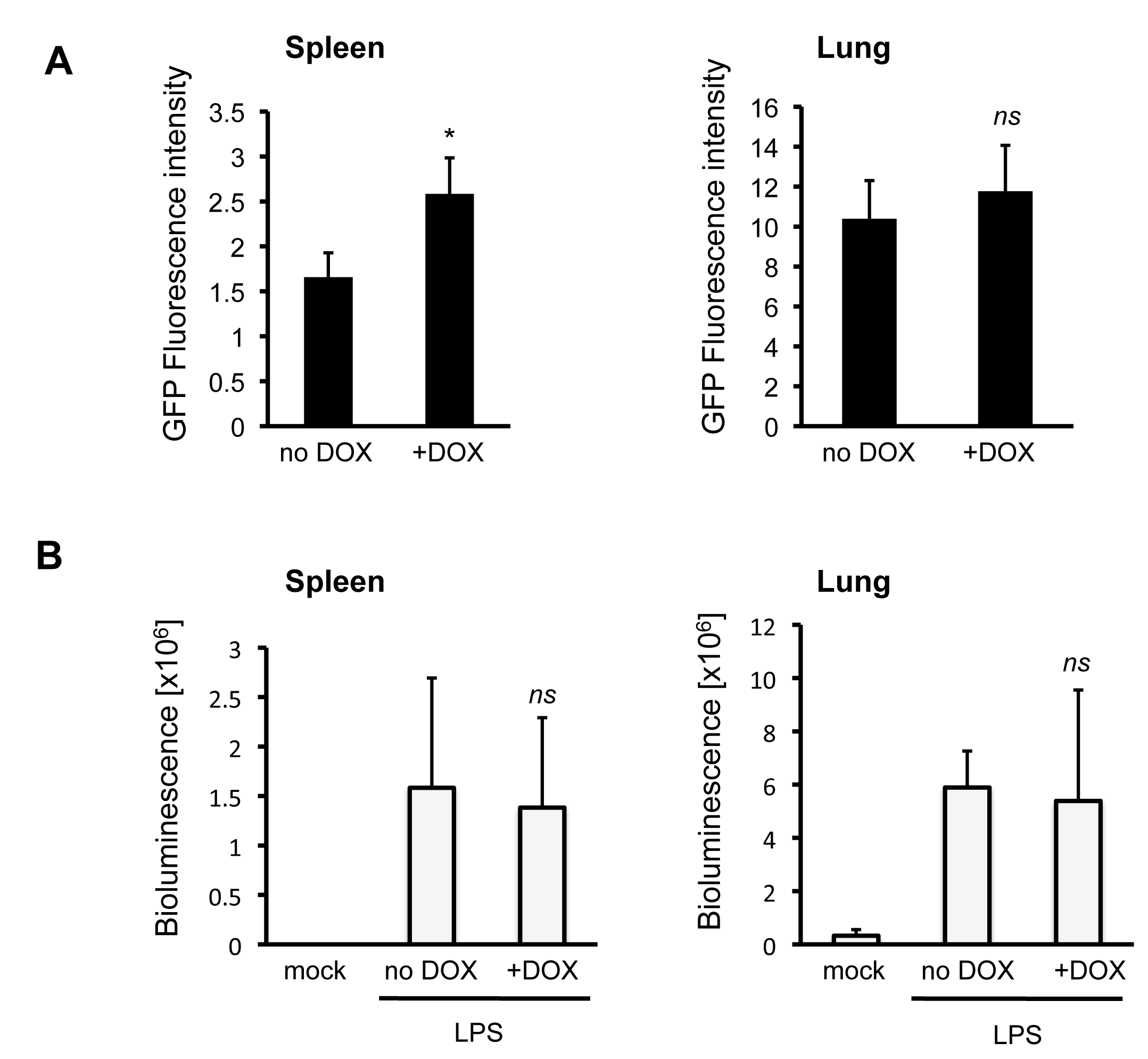

Supplement: Figure S4 — (A) GFP fluorescence imaging of spleen and lung tissue from triple transgenic shCox2/C3/Luc+ mice that received a DOX containing diet (+DOX) or a control diet (no DOX) for 12 days. GFP fluorescence is increased significantly, but not to a great extent in the spleens. In the lungs, there was no significant difference in GFP fluorescence between shCox2/C3/Luc+ mice on a DOX containing diet and on a DOX free diet. (B) Luciferase induction by interferon gamma and endotoxin (IFN-γ/LPS) in the spleens and lungs of triple transgenic shCox2/C3/Luc+ mice that were fed a DOX containing diet (+DOX) to induce shRNA expression or a control diet (no DOX) for 12 days. Mice were injected i.p. with IFNγ followed two hours later with LPS, or with saline (mock). After 6 hours, mice were euthanized, tissues were removed and luciferase bioluminescence was quantified by bioluminescence imaging. Luciferase expression was not significantly different in mice receiving the DOX diet versus controls. Data are means +/− SD. (*, p<0.05, ns, p>0.05, n = 4). (TIF) [file pone.0101263.s004.tif]
